# Scanpro is a tool for robust proportion analysis of single-cell resolution data

**DOI:** 10.1038/s41598-024-66381-7

**Published:** 2024-07-06

**Authors:** Yousef Alayoubi, Mette Bentsen, Mario Looso

**Affiliations:** 1 Bioinformatics Core Unit (BCU), Max Planck Institute for Heart and Lung Research, Bad Nauheim, Germany; 2 Cardio-Pulmonary Institute (CPI), Bad Nauheim, Germany

**Keywords:** Data mining, Data processing, Software, Statistical methods

## Abstract

In higher organisms, individual cells respond to signals and perturbations by epigenetic regulation and transcriptional adaptation. However, in addition to shifting the expression level of individual genes, the adaptive response of cells can also lead to shifts in the proportions of different cell types. Recent methods such as scRNA-seq allow for the interrogation of expression on the single-cell level, and can quantify individual cell type clusters within complex tissue samples. In order to identify clusters showing differential composition between different biological conditions, differential proportion analysis has recently been introduced. However, bioinformatics tools for robust proportion analysis of both replicated and unreplicated single-cell datasets are critically missing. In this manuscript, we present Scanpro, a modular tool for proportion analysis, seamlessly integrating into widely accepted frameworks in the Python environment. Scanpro is fast, accurate, supports datasets without replicates, and is intended to be used by bioinformatics experts and beginners alike.

## Introduction

Understanding the response of complex tissues and cells to influxes of environmental signals is one of the aims of research in higher organisms. Individual cells respond to perturbations by regulation of gene expression, which can increase the heterogeneity of individual cells within cell types, leading to different phenotypes. However, besides changes in the transcriptome within cell populations, processes such as proliferation, apoptosis, cell migration and differentiation can lead to changes in the composition of cell types within a given tissue. Such shifts of cell type proportions have been reported in various scenarios. For instance, compared to younger controls, supercentenarians—people who are aged 110 years and over—showed an increased level of cytotoxic CD4 T cells compared to younger controls^1^. In the context of COVID-19, patients exhibited differences in composition and counts of CD8+ T cells between moderate and severe cases^2^. Moreover, specific cell populations sometimes occur exclusively within individual experimental conditions, such as seen during the skeletal muscle regeneration process in mice, where non-injured fibro-adipogenic progenitors (FAP) cells differentiate into a population of activated FAP cells with a distinct expression profile^3^. These exemplary findings suggest that not only changes in gene expression within individual subpopulations of cells, but also shifts in the proportions of cell types are important during biological processes and for disease progression.

There are different methods available to investigate cell proportions between conditions. One imaging based option is to use immunohistochemistry or immunofluorescence staining to label specific cell types within the tissue sample of interest, with subsequent cell counting^4,5^. However, recent technical advances in cell capturing methods such as microfluidic droplet-based methods^6^ have introduced single-cell resolution sequencing technologies on the transcriptional (scRNA-seq) or chromatin accessibility (scATAC-seq) level^7,8^. Through sequencing and subsequent bioinformatics analysis, single-cell sequencing allows for the annotation of known cell types by comparing e.g. gene expression of individual cells to established marker genes, but also supports the identification of novel cell subtypes within complex cell mixtures^9^. One of the most commonly used tools for analysis of single-cell data is the Python-based Scanpy toolkit, which saves data matrices into a format known as AnnData, which enables preprocessing, cell clustering and marker gene assignment within one framework^10^.

Based on the clustering of single-cell resolution data into cell types, differential compositional analysis, which we define as proportion analysis (PA), can be performed. Due to technical (e.g. sequencing depth, bias in the cell extracting method and different cell decay rate) and biological (e.g. differences in age) effects, raw cell counts cannot be compared directly between clusters. Instead, more sophisticated statistical analyses are needed to gain reliable results. Recently, a number of different approaches for PA and abundance testing have been introduced, including propeller^11^, scDC^12^, dirichlet regression^13^, sccomp^14^ and scCODA^15^, as well as a method that utilizes partially overlapping neighborhoods on a *k*-nearest neighbor graph (MILO)^16^. One of the tools outperforming earlier implementations is the R tool propeller^11^. It uses a linear regression approach, followed by empirical Bayes statistical testing to mitigate the effect of sample-to-sample variance. In addition, the method allows for different data transformations, e.g. using logit and arcsin square root functions. However, a striking limitation of this method is the dependency on replicates for all conditions investigated. Due to the high cost of experiments, replicated single-cell datasets are rare, and the application of propeller is therefore limited to few datasets. Another recent package utilizing a Bayesian model is scCODA^15^, which does not necessarily require replicates in order to estimate differential composition, by utilizing appropriate priors to accommodate for the low-replicate scenario in single-cell RNAseq data. In this context, the scCODA model uses a reference cell type to refer to changes in abundance of other cell types with respect to this reference. scCODA achieves this by forcing all effects on the reference cell type to be zero. As the manual selection of a reference cell type can be challenging, the scCODA tool provides an automated reference selection.

In order to extend the field of PA approaches with a modular tool seamlessly integrating into the widely accepted Python frameworks such as Scanpy and able to natively handle anndata objects, we here present Scanpro (Single-cell analysis of proportions). Scanpro implements the linear regression approach proposed by propeller, and extends this by a bootstrapping functionality to internally simulate replicates (pseudo-replicates) from given cell distributions for unreplicated datasets.

## Results

### Package, features, and workflow

**Figure 1:**
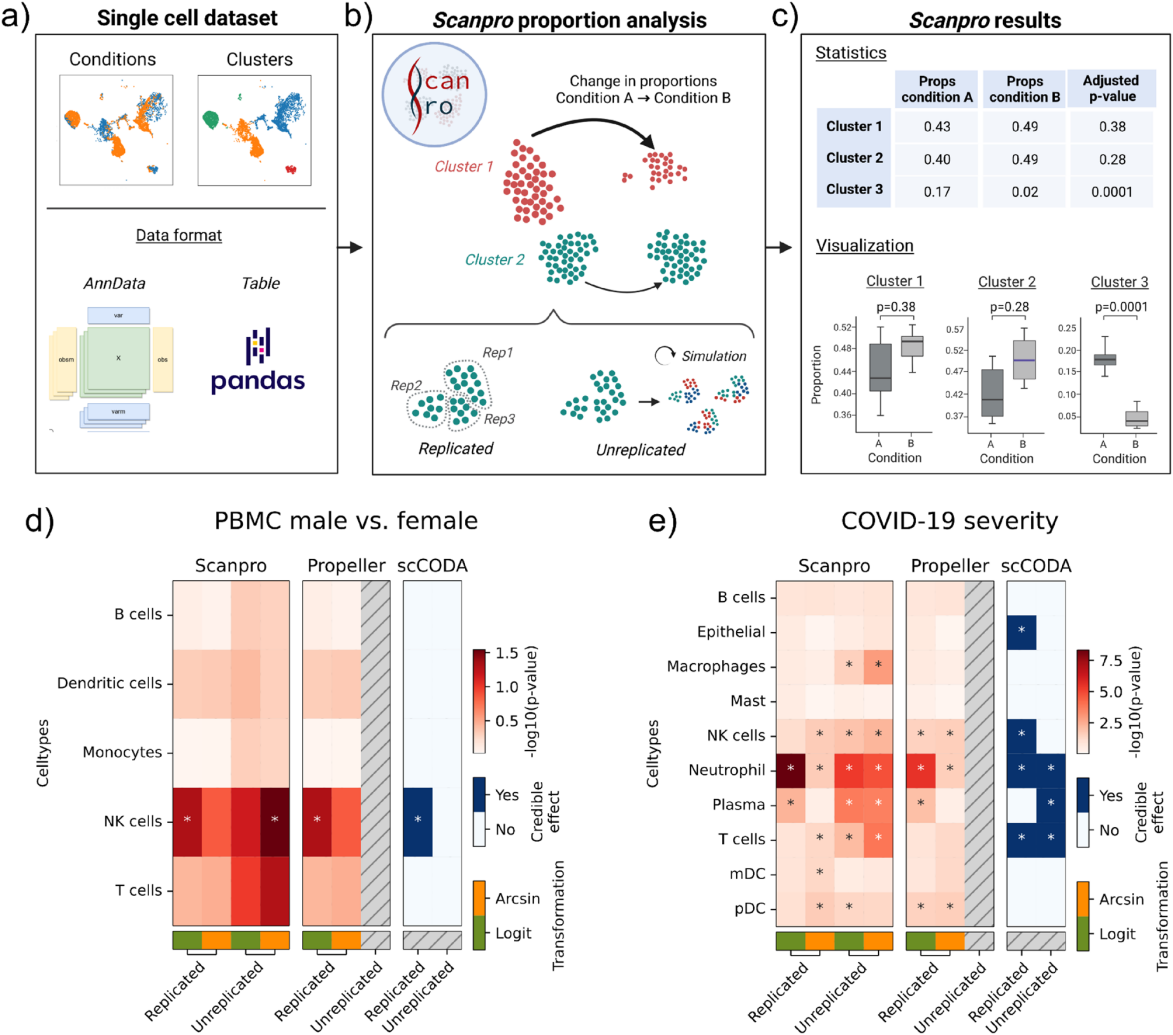
Proportion analysis using Scanpro. (**a**) Scanpro takes an AnnData object or a pandas dataframe with conditions and cluster annotation as input. (**b**) It accepts replicated and simulated pseudo-replicates for unreplicated datasets. (**c**) The results include statistics for each cluster and different visualizations of cluster composition. d,e) comparison of Scanpro with propeller and scCODA for two datasets. For Scanpro and propeller, − log10(p-value) is shown in red color scale, for scCODA’s credible effect (see^15^ for definition) is shown in blue. Arcsin or logit transformation options are available for Scanpro and propeller. Significance level for propeller and Scanpro is p < 0.05. For scCODA, PBMC data uses FDR = 0.3 and reference cell type “monocytes”; COVID-19 data uses FDR = 0.2 and reference cell type “Mast”. *NK cells* Natural killer cells, *mDC* Myeloid dendritic cells, *pDC* plasmacytoid dendritic cells. Subfigures a-c were created with BioRender.com.

Prior to PA, a standard single-cell analysis including clustering is required. After establishing the clusters and conditions to compare, Scanpro can be run on the data using the widely accepted AnnData class object and thus integrated into the Scanpy (scRNAseq), Episcanpy (scATAC), and MUON (multiomics) ecosystems in Python^17^ (Fig. 1a). In addition, a table of cells with annotations in Pandas format is supported. During the analysis, Scanpro uses the number of cells within each condition to estimate whether the cells have different composition in either of the clusters. When the data is replicated, Scanpro applies a Python implementation of the empirical Bayes method presented in the propeller tool. However, when the data is unreplicated, Scanpro offers a robust method to simulate pseudo-replicates by splitting the original samples into multiple replicates using bootstrapping without replacement, which extends the usability of the tool to non-replicated datasets (Fig. 1b). While this method cannot replicate the biological variance of real replicates, the randomized bootstrapping explores the possibilities that the observed changes in cluster sizes arose by chance. In order to control for outliers of the randomized splitting, the pseudo-replication method is run 100 times and the median p-values for each cluster are calculated. After the analysis, Scanpro reports final statistics, as well as matrices for cell proportions, experimental design, and integrated plotting methods to visualize proportions (Fig. 1c). These visualizations include a box plot overview of samples (either original or simulated), which can be used to visually confirm differences in cell proportions per cluster. Moreover, Scanpro provides the possibility to restrict the analysis to certain conditions of interest, add covariates per sample as well as support multi-condition comparison using ANOVA. Scanpro is intended to be used at various levels of bioinformatic proficiency by providing exemplary jupyter notebooks and an extended manual within the public code repository found at: https://github.com/loosolab/scanpro.

### Comparison to existing tools

In order to assess the overall performance of Scanpro in comparison to existing tools, we selected propeller and scCODA as the current state-of-the-art methods for PA. Of note, our intention here is to show overall comparability and functionality of tool features, while finding optimized parameter settings is discussed later in this manuscript. For the comparison, we applied each tool to three datasets, namely (I) A PBMC dataset with transcriptome profiles of peripheral immune cells from male and female participants from two distinct age groups (young and adult)^18^, (II) a single nucleus (sn) RNA-seq dataset profiling healthy cardiac cell types across three developmental stages (fetal, young and adult)^19^, and (III) a scRNA-seq dataset of immune cells in bronchoalveolar lavage fluid from patients with varying severity of COVID-19 and healthy individuals as a control group^2^. These datasets were also used in the propeller and scCODA papers and tutorials, and contain biological replicates, which make them ideal for comparison. To test the tools on data without replicates, we merged the biological replicates of each condition and dataset respectively, and compared the performance of Scanpro with scCODA, since propeller does not support unreplicated datasets.

For the results of scCODA, we observed that the default FDR of 0.05 was too strict to yield any significant clusters, and we thus increased the FDR to balance false negatives and false positives (Supplementary Fig. 1). Of note, we compared all tools without taking covariates into account, although all tools are able to handle covariates. When comparing the results of each tool, we found similar results between Scanpro and propeller for the (I) original PBMC dataset for male vs. female, which was expected as Scanpro is a reimplementation of propeller for data with replicates (Fig. 1d). Using logit transformation, both tools found a significant difference in natural killer (NK) cells, which is also visible in the per-cluster proportion plots provided by Scanpro (Supplementary Fig. 2). Interestingly, when run on the data on pseudo-replicates (we refer in all figures to biological replicates as replicated, and pseudo-replicates as unreplicated), we found that Scanpro was still able to detect the significant difference in NK cells using the arcsin data transformation, whereas scCODA identified the T cell cluster as significant. The original study observed a higher percentage of NK cells in male compared to female samples^18^, which confirms the results of propeller and Scanpro.

For (II) the heart development dataset, all three tools identified the same four out of eight cell types with a significant change in cell distribution between fetal, young and adult samples (Supplementary Fig. 3). The run on original data identified fibroblasts and cardiomyocytes as significantly changed only using logit transformation, whereas the unreplicated run found these to be significant regardless of transformation. For this data, both Scanpro and scCODA were able to identify significant clusters in the unreplicated data, although scCODA additionally identified neurons as differentially changed. This effect can be mitigated by lowering the FDR of scCODA to 0.1, but this causes loss of most other significant clusters. (Supplementary Fig. 1).

Lastly, we ran the tools on III) the COVID-19 dataset, which compares cell proportions between healthy individuals and COVID-19 patients with moderate or severe outcomes. Liao et al. found that patients with severe COVID-19 had a distinct immune cell profile compared to those with moderate outcome, namely an increase in macrophages and neutrophils, and a decrease in T cells, myeloid dendritic cells (mDCs) and plasmacytoid dendritic cells (pDCs)^2^. By performing PA, we found a number of differences in the results of the tools (Fig. 1e). Firstly, we found minor differences in the results between Scanpro and propeller on the replicated data, as Scanpro assigns T cells and mDCs as significant (p = 0.049), whereas these are just slightly above the demanded p-value in propeller (p = 0.054). These differences might arise due to different implementations of e.g. linear regression method between Python and R. Interestingly, the T cells are also assigned as significant by scCODA, and were highlighted in the original publication, suggesting that this cluster should indeed be assigned as significant. When running the tools without replicates, we saw that Scanpro could reproduce the results from either logit or arcsin runs with replicates with the exception of the mDCs. Of note, Scanpro instead found changes in the number of macrophages across COVID-19 severity. While this was not found by either propeller or scCODA, the authors of the dataset mentioned a change in macrophage proportions between moderate and severe COVID-19 samples, but could not show significant changes using a normal T-test due to large variance in the healthy control samples (Supplementary Fig. 4). A shift of macrophage composition was also reported for COVID-19 patients in another study^20^. Thus, while PA on the original data did not call the cluster as significant, the pseudo-replication on the combined dataset highlighted the potential change in macrophage composition (Supplementary Fig. 5).

In summary, we compared our tool Scanpro on well described replicated test datasets to two existing state-of-the-art tools. In this context, Scanpro provides the first Python implementation of a linear regression approach to perform PA, which works both with and without replicates, while providing highly comparable conclusions to given tools.

### Performance on simulated datasets

Next we assessed the robustness of our Python implementation and the influence of parameters such as the transformation method for stabilizing variances, the pseudo-replicate number chosen for the PA if biological replicates are missing, and the influence of the quantity of a cell type within a dataset (signal strength). In order to do so, we performed another benchmarking test with simulated datasets. Cell proportions were simulated following a hierarchical model as described in the propeller paper^11^, where cells for each replicate were drawn randomly from a negative binomial distribution. As suggested by the authors of propeller, two types of tests were carried out:

I. A null simulation test, where two conditions and five clusters were simulated, with the proportions not changing significantly across conditions.
II. A proportion test where three out of seven cell types are changed, based on differences and overall cell proportions derived from a real dataset^19^.

For both tests, we generated various numbers of replicates ranging from 2 to 14 with 100 datasets each, while holding the total number of cells at around 20,000 cells per condition, summing up to 1400 test datasets (Supplementary Fig. 6). To assess significance, we chose significance level at 0.05. For each number of replicates, we calculated the accuracy values, defined as the ratio of correctly assigned clusters (changed as true positives and not changed as true negatives) and the number of all clusters within the 100 simulations.

**Figure 2:**
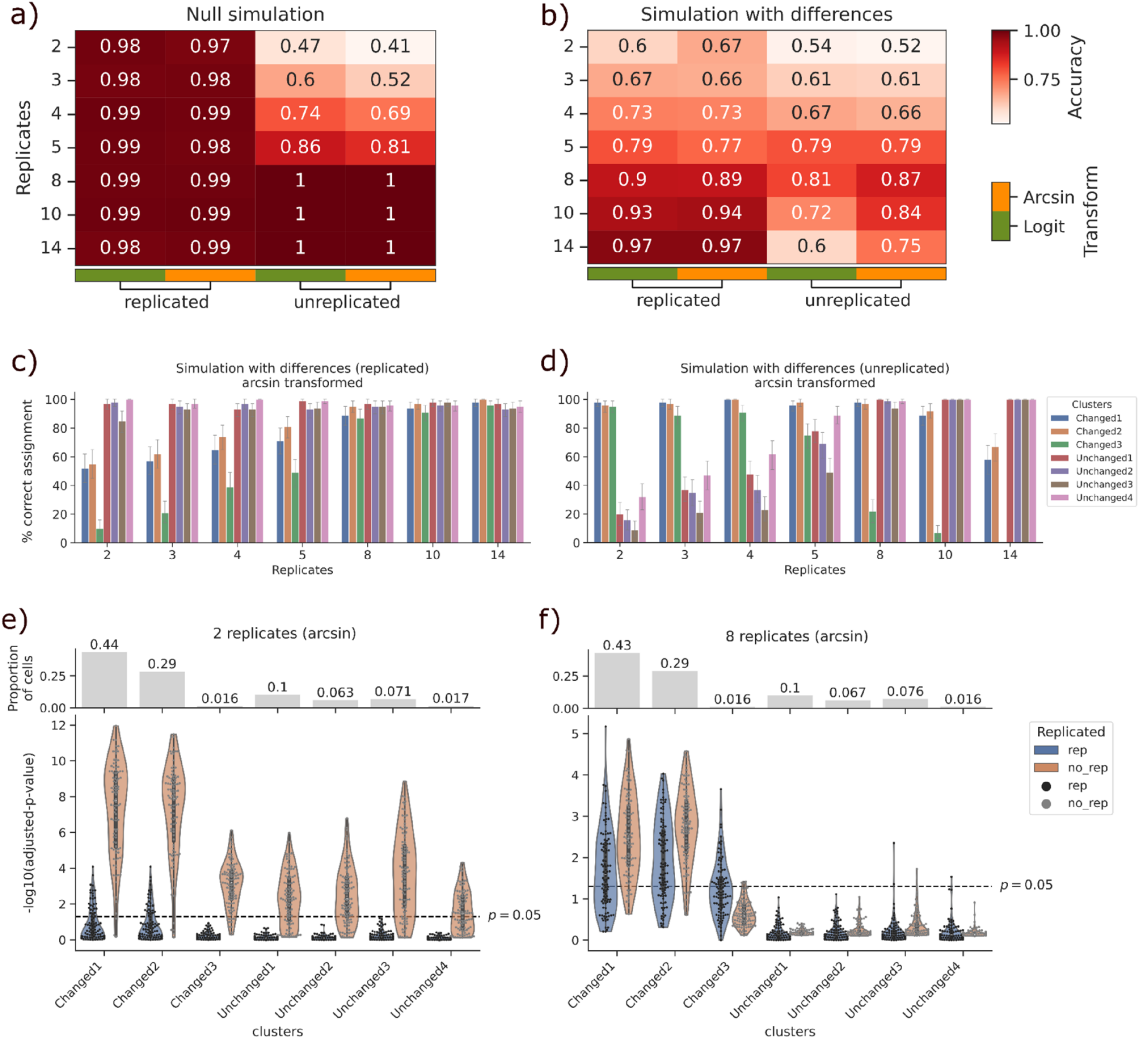
Results of normal and bootstrapping methods on simulated datasets. (**a**, **b**) Accuracy values for two simulations; one without differences (**a**) and one with differences in three of seven clusters (**b**). Accuracy is calculated over 100 simulations. Method of transformation is abbreviated “transform”. (**c**, **d**) Percentage of correct assignments for each cluster in simulation with differences for both replicated (**c**) and unreplicated (**d**) datasets, each bar represents 100 assignments. (**e**, **f**) Distribution of p-values per cluster for all 100 runs with four (**e**) and 14 (**f**) original (rep) and pseudo-replicates (no_rep). Replicates are abbreviated as reps, the dashed line in (**e**, **f**) shows the − log10(0.05) significance level. Number of cells per condition is ~ 20,000.

In I) the null simulation on replicated data, Scanpro had nearly a perfect accuracy (0.98–0.99, Fig. 2a, Supplementary Fig. 7a). Of note, these high accuracy values also applied for the Scanpro unreplicated run with pseudo-replicates of eight and above. For lower number of pseudo-replicates (two to five), we observe worse accuracies than for replicated runs. The accuracy values are increasing with the number of pseudo-replicates, but perform worse compared to the same number of real replicates. Interestingly, the unreplicated data with higher pseudo-replicate numbers provided slightly better accuracies than the replicated data, which can be explained by the simulation of pseudo-replicates, which will naturally lack some of the noise found in the original replicates. Importantly, we did not observe significant differences between logit and arcsin data transformations. Thus, these results indicate a good control of false positives for datasets with and without replicates.

In the (II) proportion test, we observed high accuracies (0.6–0.97) for Scanpro runs with replicates for both arcsin and logit transformations as expected (Fig. 2b). However, for the run without replicates, we observed a difference between arcsin and logit transformation, with arcsin slightly outperforming logit in datasets with number of replicates from 5 to 14. More importantly and in contrast to the run with replicates, we observed large differences in accuracy in dependency of the number of pseudo-replicates. For the 20,000 cells investigated per condition, we observed a sweet spot around 5–8 pseudo-replicates for the accuracy, while it decreased with more or less pseudo-replicates.

In order to further investigate the low accuracy values in dependency of smaller and larger numbers of pseudo-replicates, we looked at the correct assignment rates for each cluster in the test II data individually. For replicated datasets, we see an overall increase of true positives with more replicates, while the percent of correct assignments for unchanged cell types remains nearly perfect for all numbers of replicates (Fig. 2c). Only one changed cluster (Changed3) indicates the need for a certain number of replicates (Fig. 2c, green bar).

In contrast, for unreplicated datasets, the percent of correct assignments for all clusters peaks at 8 pseudo-replicates, while differences on the correct assignment rate for individual clusters can be observed (Fig. 2d). Of importance, we observed the same cluster “Changed3” with changes in the correct assignment rate, but this time with good performance for low pseudo-replicate numbers up to 4 and then decreasing with higher pseudo-replicate numbers (Fig. 2d).These results are consistent for logit transformed data (Supplementary Fig. 7b).

For performance comparison reasons, we also benchmarked scCODA with the unreplicated datasets for both tests. While scCODA scores nearly perfect in the null test, the accuracies for the test with differences fell behind the performance of Scanpro (Supplementary Fig. 8).

In order to further explore this observation on the Changed3 cluster (Fig. 2c+d), we looked at the p-values per cluster for two representative replicate numbers (2 and 8) for replicated and pseudo-replicated datasets respectively (Fig. 2e+f). In this context, we found obvious differences in the overall p-value distribution for individual cell clusters in dependency of the number of replicates, with a more prominent separation for the higher pseudo-replicate number. Of note, pseudo-replicates per se tend to generate a higher false assignment rate compared to real replicates when comparing with the true state of the clusters (changed/unchanged). For the two replicates, we see slightly inflated significance for all clusters using the bootstrap mode for unreplicated dataset, while the replicated mode struggles with finding significance for the “Changed3” cluster only (Fig. 2e). When increasing the number of replicates to 8, Scanpro is able to find all significant clusters from the replicated data, whereas the bootstrap mode only fails to find significance for “Changed3” (Fig. 2f). Along this line, we postulate a high correlation of the strength of the separation with the number of cells assigned to the respective cluster (Fig. 2e+f from left to right). Changed3 comprises less than 2% of the total number of cells in the dataset, which can explain the failure to identify it as significant when sampling it into smaller parts.

In conclusion, we found that the Scanpro proportion analysis provides robust results for replicated data, while the bootstrapping method for pseudo-replicates introduced by Scanpro is a versatile approach to deal with unreplicated datasets. In this context, we found evidence that the total cell number of a dataset and the given cell composition is of importance for optimal parameter choice in PA. In general, PA on very small cell clusters harboring less than 2% is hard to perform and should be avoided by favoring lower resolution clusterings, or by leaving small cell clusters out of the analysis.

### Optimizing bootstrapping parameters by observed cell counts

**Figure 3:**
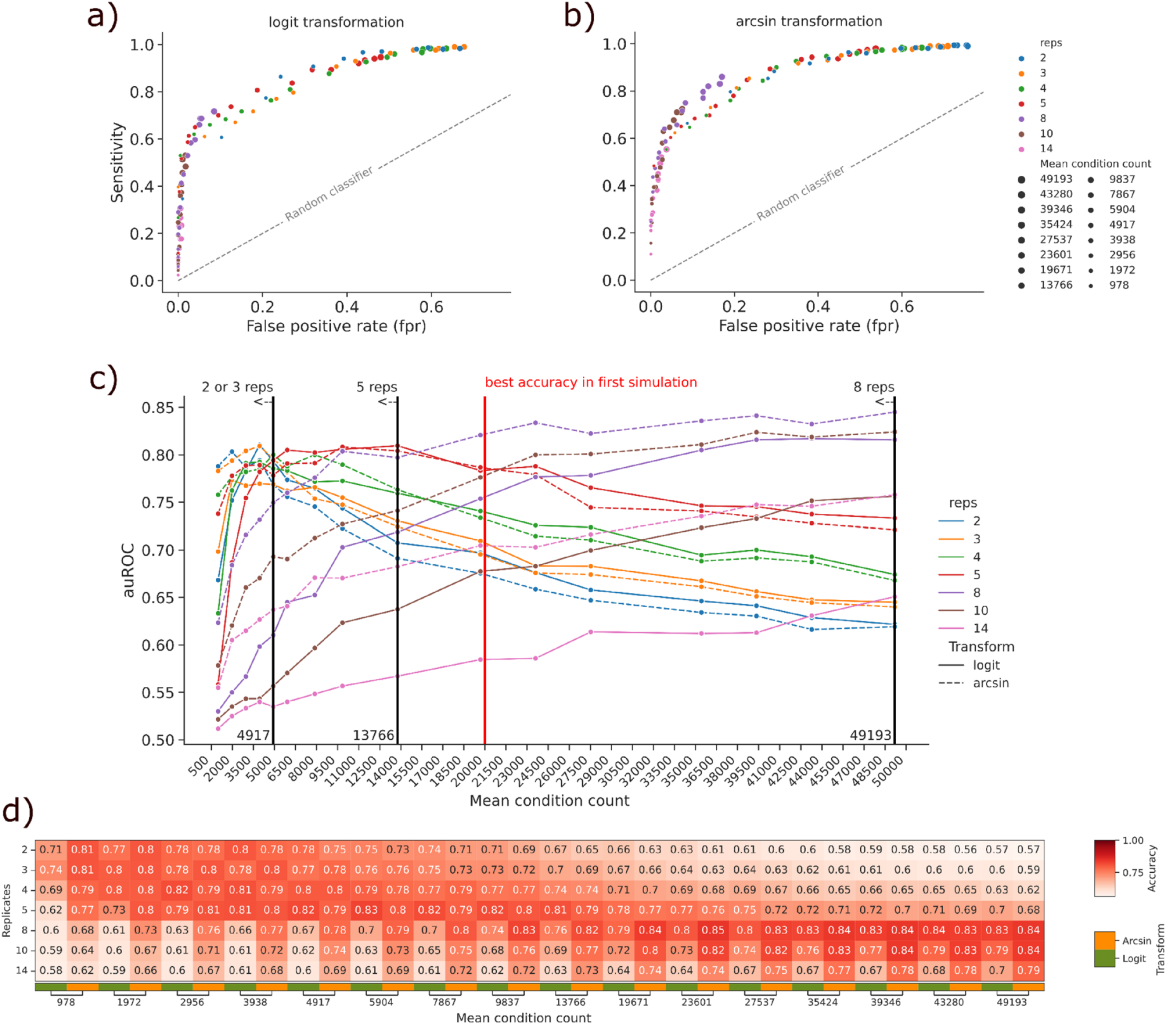
Benchmarking Scanpro bootstrapping with increasing cell counts. (**a**, **b**) Sensitivity vs. false positive rate (FPR) for simulated runs using logit and arcsin transformation respectively. (**c**) Mean cell counts vs area under the ROC curve (auROC) for each combination of pseudo-replicates and transformation. Number of pseudo-replicates are abbreviated as reps; colored curves indicate individual number of pseudo-replicates; type of transformation is shown as solid or dashed line; vertical lines indicate number of pseudo-replicates with the best auROC for three ranges of mean cell counts. (**d**) accuracy values for each combination of pseudo-replicates and transformation.

The previous simulations showed significant differences in results based on the number of pseudo-replicates generated by the bootstrapping method. Since the number of cells per condition was kept stable, we suspected an effect of cell count per pseudo-replicate on the performance of Scanpro. Thus, in order to systematically assess the relationship between the total cell count within an experiment and the number of pseudo-replicates to create by bootstrapping, we performed additional simulations with increasing total cell counts per condition (16 steps from ~ 1000 to ~ 50,000 cells). As before, 100 simulations were run for each iterative cell count, generating 100 datasets with 3 cell types out of 7 with significant changes in proportions. For each simulated dataset, the bootstrapping method was applied with (2, 3, 4, 5, 8, 10, 14) pseudo-replicates each and both “logit” and “arcsin” transformations (14 runs per dataset). For each combination the false positive rate (FPR) and true positive rate (sensitivity) were calculated (Fig. 3a+b). As expected, we found the FPR to increase with fewer pseudo-replicates, while the sensitivity decreases with more pseudo-replicates. In order to determine an optimal default value for the number of pseudo-replicates to be chosen in dependency of a total cell count in an experiment, we calculated the area under the ROC curve (auROC) for each mean count per condition (Fig. 3c). Interestingly, the auROCs for each run indicate good accuracies for 2 and 4 pseudo-replicates for smaller mean cell counts (up to 5000 cells), and dramatically lower accuracies for larger mean cell counts (Fig. 3c). Accordingly, 8, 10 and 14 pseudo-replicates performed better with datasets having more than 25,000 cells. For smaller numbers of pseudo-replicates, the influence of the transformation method with cell counts > 5000 is negligible, whereas we saw better results for arcsin transformation with larger numbers of pseudo-replicates and cell numbers > 5000. Moreover, we calculated the accuracy for each combination of pseudo-replicate and mean condition count (Fig. 3d). We can clearly see that as the mean condition count increases, accuracies improve for bigger numbers of pseudo-replicates.

Summarizing, we found that the total number of cells of a single-cell dataset has great influence on the results of PA when forced to simulate pseudo-replicates due to the absence of biological replicates. However, when selecting the appropriate number of pseudo-replicates dependent on the given number of cells, we found Scanpro to provide a robust method, which significantly increases the operational area of PA to unreplicated data.

### Performance on human cell atlas data

Next, we investigated the performance of Scanpro to analyze large-scale single-cell datasets across several tissues and cell types. Therefore, we obtained two datasets: (I) A part of the human cell atlas project^21^, where Kuppe et al. presented a spatial multi-omic map of human myocardial infarction (MI)^22^, and (II) an scATAC-seq dataset of multiple fetal tissues from the human cell atlas of fetal chromatin accessibility^23^. Using these data we ran Scanpro on both replicated and unreplicated data by merging all biological replicates per condition. To evaluate the outcome, we compared our findings to the findings described in the respective manuscripts.

**Figure 4:**
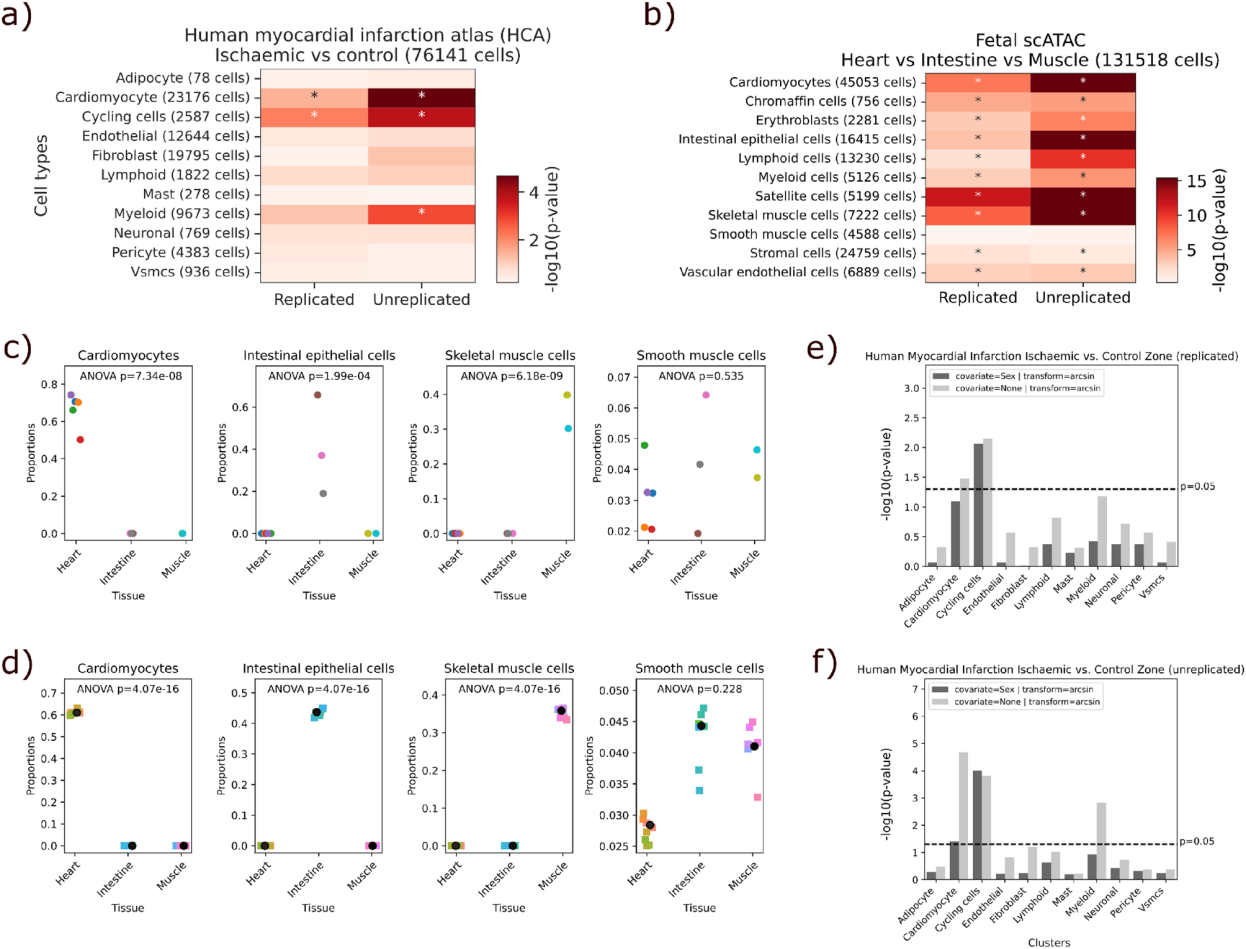
Scanpro analysis of large single-cell atlas datasets. (**a**, **b**) Scanpro results of Human myocardial infarction atlas and Fetal scATAC datasets with and without replicates, with arcsin transformation. Values are − log10(p-values), asterisks indicate significant change (p < 0.05), for unreplicated runs, 8 pseudo-replicates were set. (**c**) Original fetal scATAC sample proportions for specific cell types. (**d**) Fetal scATAC pseudo-replicates proportions generated by Scanpro’s bootstrapping method. Black dots are the mean replicate cell proportions; colored squares are cell proportions of pseudo-replicates. (**e**, **f**) results of the Human myocardial infarction dataset with age as condition and sex as covariate, with biological replicates (**e**) and pseudo-replicates (**f**).

In the case of (I) human MI, the study used various techniques such as snRNA sequencing, snATAC sequencing, and spatial transcriptomics to create a comprehensive understanding of the molecular changes that occur during a heart attack, providing a resource map of the human heart at early and late stages after MI compared to control hearts. The study includes the analysis of different zones in the human heart during and after MI. One of the analyzed zones is the ischemic zone, which refers to the area of the heart that has been affected by a MI. We used Scanpro to analyze the cell type proportions between ischaemic and control snRNA-seq samples (Fig. 4a, see Supplementary Fig. 9 for scCODA results). Besides cardiomyocytes and cycling cells, our bootstrapping method found myeloid cells to have significantly changed (Supplementary Figs. 10 and 11). Interestingly, the original publication reports all three cell types to have significant abundance changes between ischaemic and control samples^22^. Of note, two of these three cell types with proportion changes have comparable high cell counts, while cycling cells are in the critical range of 1–2% of the total cells. Other small cell populations such as adipocytes and mast cells did robustly not show a proportion change. Next, we analyzed the ischemic and control samples based on pairwise combinations of the individual replicates (instead of merging all replicates into one) to address overall small cell numbers and variation between individual samples. We chose three control and six ischemic samples (Supplementary Fig. 12a) and ran Scanpro with 18 different pairs of replicates (Supplementary Fig. 12b). Same analysis was done for scCODA, while Scanpro resulted in better performance (Supplementary Fig. 12c).

The (II) scATAC-seq data originates from the “human cell atlas of fetal chromatin accessibility”, which is an ambitious project to map and document open chromatin regions across the genome^23^. This data provides chromatin accessibility profiles of single cells across multiple fetal tissues, as well as assignment of specific cell types. While different tissues can share cell types with other tissues, there are also many tissue-specific cells. Hence, PA on such data will highlight both tissue-specific cell types and significant changes in cell types shared across tissues. To investigate this effect, we ran Scanpro on a subset of the dataset containing heart, intestine and skeletal muscle samples (Fig. 4b). As expected, the results show significant changes in specialized cells such as cardiomyocytes, intestinal epithelial cells and skeletal muscle cells, which were consistent for runs both with and without replicates (Fig. 4c,d; Supplementary Fig. 13a,b). Interestingly, smooth muscle cells showed no significant changes, suggesting they are shared between all three tissues.

In conclusion, we find that Scanpro is applicable to large-scale single-cell datasets, both in the context of disease states, but also in quantifying differences in cell type composition between tissues. Both datasets have comparable high cell counts per cluster, making the analysis with bootstrapping more robust.

### Performance with covariates

Scanpro can also take covariates into account when performing PA. In this context, we re-analysed the human MI dataset presented above (comprising scRNA samples from control and ischaemic patients), this time adding sex as a covariate, once using biological replicates and once merging these replicates and generating pseudo-replicates with Scanpro (Fig. 4e,f). Interestingly, we found that while the proportions of cycling cells were not affected by sex, the significant changes within cardiomyocytes and myeloid cells (Fig. 4a), are partially driven by the sex covariate. Further, we analyzed the PBMC composition from the dataset used in the tool comparison section by age, and found significant changes in monocytes between young and old individuals. Adding sex as a covariate to this analysis had no effect on significance of monocytes (Supplementary Fig. 14, left). However, when analyzing the composition of cells by sex, we found that adding age as a covariance uncovered a significant difference in the proportions for NK cells (Supplementary Fig. 14, right). This shows that the influence of covariates can be significant to the results of PA and should be adjusted for where appropriate.

## Discussion

PA is a method used in cell biology and pathology to estimate the relative abundance of different cell types between conditions within a tissue of interest. This type of analysis is particularly useful in studies on conditions that affect multiple cell types, such as cancer or inflammatory disorders^24,25^. Traditional omics approaches such as RNA-seq or ATAC-seq rely on bulk tissue samples and often mask these important cell-to-cell variations in gene expression and cell type composition. Although deconvolution approaches exist that try to predict cell identities from bulk data^26,27^, single-cell analysis techniques provide a means to study cellular heterogeneity in more detail and at higher resolution. In this context, there is an urgent need for robust computational tools to analyze and interpret single-cell resolution datasets. In this manuscript, we introduced Scanpro as a versatile tool to perform PA in a highly automated fashion, which is able to handle input from Python AnnData objects and thus permit integration into standard analysis frameworks in the Python universe.

In the single-cell sequencing context, PA relies on certain assumptions that can lead to significant misinterpretations if not handled specifically. For example, correct cell clustering and thus cell type identification is paramount for PA. Classically, unsupervised clustering is calculated on the basis of a neighborhood graph, which connects each cell with its most similar neighbors. As a direct consequence, the parameters of the algorithm, such as resolution for Leiden clustering, has a critical effect on the number and size of resulting clusters. In order to achieve biologically meaningful PA, clusters should represent real cell types, however, this is a challenge for the pre-processing steps prior to PA. In addition to clustering itself, there are challenges associated with technical and biological variability, as well as data sparsity. Technical variability could arise from the experimental design itself. For instance, utilizing different sequencing machines for replicates or even collecting samples at different timepoints may introduce batch effects that lead to artificially introduced changes in genomic features, thus highly impacting the clustering results^28^. Along this line, clusters might not necessarily reflect cell types, but are potentially defined by these technical parameters as well. Thus, care should be taken when selecting clusters and interpreting PA results.

We introduced a bootstrapping method with Scanpro to extend the functionality to include unreplicated datasets. However, as indicated by the benchmarking test and the real datasets, bootstrapping has limited benefits when handling rare cell types. From the benchmarking, we found clusters supported by less than 2% of all cells in an experiment to produce unstable results. This aspect has to be taken into account when choosing parameters for cell clustering, and we strongly recommend generating larger, less specific cell clusters with higher cell numbers rather than producing a large number of potentially highly specific cell types with small cell numbers each. Aside from these technical issues, it is noteworthy to mention that the lack of replicates limits our ability to draw definitive conclusions about the variability in the data and per se is not advisable. However, as a large number of publicly available SC experiments do not provide replicates, we address this issue by our iteration-based bootstrapping method. Although this approach allows for proportion analysis on non replicated datasets and often results in the same conclusion as replicated datasets (see benchmarks), the results should be seen as an indication of cell types that have a proportional shift and should be interpreted with caution and additional experimental validation.

In conclusion, Scanpro is a robust tool to perform PA on single-cell data. It overcomes limitations of other tools which need replicated datasets or a reference cell type, and offers integrated visualization for direct investigation of cell proportions. With its easy integration into existing frameworks in the Python environment, Scanpro can serve as a default step in any single-cell pipeline, and can help to identify differential proportions in a variety of different datasets.

## Methods

### Transform proportions

Scanpro starts by transforming the cell counts per cluster and replicates into a proportions matrix, where rows are the replicates and columns are the clusters/cell types. The matrix consists of cell proportions of each cluster/cell type in each replicate so that each row sums to one. Since clusters’ proportions are binomially distributed, which makes the variances heteroskedastic, stabilizing the variances is an important preprocessing step. Scanpro supports two possible transformation methods; (a) logit transformation log(proportions/(1 − proportions)) and (b) arcsin square root transformation arcsin(sqrt(proportions)). According to the developers of propeller, the arcsin transformation works better for higher numbers of replicates or when there are outliers in the data^11,29^.

### Linear modelling

Linear modeling offers an easy, straightforward approach to testing the relation between a set of independent observations and a dependent variable. In the field of genomics, linear modeling has been widely used in assessing differentially expressed genes. One of the more famous tools used for differential expression analysis is LIMMA, a package originally developed to process microarray data and next-gen sequencing RNA experiments^30^. The LIMMA method fits a linear model to each gene in the whole dataset, which takes into account the gene-wise variation across replicates.

To assess differential cell composition in clusters, Scanpro uses the LIMMA approach by fitting a linear model to each cluster rather than to each gene. Besides the transformed proportions matrix, the {lm_fit} function takes a design matrix as input, which can also include covariates. Scanpro uses the implementation of an ordinary least squares regression (OLS) model by statsmodel {statsmodels.api.OLS}^31^. To keep the re-implementation consistent with propeller, sigma and standard deviation values are calculated in Scanpro’s implementation similar to LIMMA’S {lmFit} function.

### Empirical Bayes statistics

Scanpro utilizes the Empirical Bayes method, which is also a function of the LIMMA package^30^. As mentioned above, variances in cell counts (similar to RNA counts) in different replicates pose a statistical challenge. These variances arise either biologically e.g. due to differences in response to different treatments, or technically, due to e.g. biases in cell extraction assays or different rates of depletion of different cell types. These variances prohibit meaningful comparisons of raw counts and should be taken into account in every downstream analysis. Using the whole dataset, the empirical Bayes method estimates a posterior variance and “shrinks” the estimated cluster-wise variances toward the estimated prior variance^32^. This allows for more robust results and mediates type I error, which leads to significantly fewer false positives.

To test significance, Empirical Bayes moderated T-test (for two conditions) and ANOVA (for more than two conditions)^32^ are used within Scanpro. Estimated p-values are then adjusted for multiple testing using the Benjamini–Hochberg method^33^.

### Bootstrapping method to simulate replicates

In order to simulate pseudo-replicates from one replicate with a similar variance between replicates as introduced by original biological replicates, Scanpro uses bootstrapping to split one replicate into multiple pseudo-replicates. After finding the replicate with the minimum number of cells (n_min) across all conditions, all other replicates are reduced to this minimum to avoid effects of large differences of replicate sizes. The reduction is done by randomly choosing n_min cells from each replicate. Next, depending on the parameter n_reps, each replicate per condition is split into n_reps pseudo-replicates. The cell count for each pseudo-replicate (n_rep) is chosen randomly (from range 0 to n_min), and n_rep cells are drawn randomly from the replicate using bootstrapping without replacement, meaning every cell is chosen once and removed from the pool. The n_min is then subtracted by n_rep and the process is repeated. This ensures the preservation of cluster proportions while also introducing some variance due to the randomness of bootstrapping. To stabilize results, the method performs 100 iterations and runs Scanpro on each generated dataset with pseudo-replicates. The beta coefficients of linear models from 100 simulations are pooled together using Rubin’s rule^34^, to give mean estimates of the proportions of each cluster. Moreover, the median values of the adjusted p-values are calculated as a final estimation, since it was shown that the median of multiple p-values from testing on multiple replicated datasets is a reliable estimate^35,36^.

### Visualization

The ScanproResults object offers two plotting methods: ScanproResults.plot() and ScanproResults.plot_samples(). The {plot} method can be used to plot the proportions of each cluster in each condition, making it easier for the user to interpret. Plots in the {plot} function are generated using the seaborn package^37^. Plots are annotated with the corresponding p-value for each cluster. The package {statsannotation} was used to annotate plots with p-values^38^.

### Benchmarking simulations

The datasets for the benchmarking were simulated using the hierarchical model described in the propeller paper^11^. The model assumes that:

I. The total number of cells for each replicate (n_j) is drawn from a negative binomial distribution.
II. The proportions for each cell type in each replicate (p_ij) is drawn from beta distribution with parameters a and b.
III. The cell counts for each cluster in each replicate are drawn from a binomial distribution with probability p_ij and dispersion (n) = n_ij.

For the benchmarking dataset shown in Fig. 2, we have set out to use ~ 20,000 cells per condition for all numbers of replicates or pseudo-replicates. In order to generate one condition with one replicate, the default parameters for the negative binomial distribution are mean cell number as (mu) = 20,000 and dispersion (n) = 20. Accordingly, for each predefined number of replicates per condition (n_reps = 2, 3, 4, 5, 8, 10 and 14), we divide the mu parameter by n_reps, which gives us less cell count per replicate but roughly the same number of cells per condition regardless of number of replicates. Using this method, we simulated 100 datasets for each n_reps. To simulate the same signal background for the bootstrapping method and pseudo-replicates, we merge the prior given replicates to have only one replicate per condition (with roughly 20,000 cells). Using the bootstrapping without replacement, each condition was split again into n_reps pseudo-replicate per condition. Thus we guarantee that for each condition, the number of cells is similar and comparable across all simulation tests.

For the optimizing bootstrapping parameters section we used the same simulation method. Here, we fixed the number of replicates per condition to four and iteratively increased the mu parameter (ranging from 250 to 12,500). We then merged the replicates and performed the bootstrapping method according to the investigated classes of 2–14 pseudo-replicates. For each mu parameter, we simulated 100 datasets.

For both tests, we use a significance level of 0.05, based on the adjusted p-values. All benchmarks are assessed by accuracy, defined as (true positives assigned + true negatives assigned) / total number of clusters.

### Supplementary Information

Supplementary Information.

## Data Availability

The PBMC data is available from the National Genomic Data Center (HRA000624)^18^. The cell proportions table was extracted from the supplementary data and processed to be used for Scanpro. The heart development data is available on Gene Expression Omnibus via accession number GSE156703^19^. The COVID-19 dataset is available via the accession number GSE145926^2^. The cells metadata file for the human fetal cell atlas scATAC dataset is available via the accession number GSE149683^23^. The processed dataset for the human myocardial infarction atlas used in this paper (All-snRNA-Spatial multi-omic map of human myocardial infarction) is available on https://cellxgene.cziscience.com/collections/8191c283-0816-424b-9b61-c3e1d6258a77, raw data can be found at https://data.humancellatlas.org/explore/projects/e9f36305-d857-44a3-93f0-df4e6007dc97. Results of all PA runs as well as benchmarking analysis are found in supplementary data.

## Code availability

The Scanpro software and detailed information on dependencies, installation and usage are available at github (https://github.com/loosolab/scanpro). Scanpro can be installed via pip or directly from source (see manual). Analysis and benchmarking code generated for this manuscript are available in the github repository within the folder “Alayoubi_et_al_2024”. ScanPro is also available within a complete SC analysis workflow via the sc_framework (https://github.com/loosolab/SC-Framework, folder general notebooks^39^).

## References

[1] Hashimoto K (2019). Single-cell transcriptomics reveals expansion of cytotoxic CD4 T cells in supercentenarians. Proc. Natl. Acad. Sci..

[2] Liao M (2020). Single-cell landscape of bronchoalveolar immune cells in patients with COVID-19. Nat. Med..

[3] Oprescu SN, Yue F, Qiu J, Brito LF, Kuang S (2020). Temporal dynamics and heterogeneity of cell populations during skeletal muscle regeneration. Science.

[4] Lee C-W (2020). Multiplex immunofluorescence staining and image analysis assay for diffuse large B cell lymphoma. J. Immunol. Methods.

[5] Blom S (2017). Systems pathology by multiplexed immunohistochemistry and whole-slide digital image analysis. Sci. Rep..

[6] Zhou W (2021). Microfluidics applications for high-throughput single cell sequencing. J. Nanobiotechnol..

[7] Buenrostro JD (2015). Single-cell chromatin accessibility reveals principles of regulatory variation. Nature.

[8] Haque A, Engel J, Teichmann SA, Lönnberg T (2017). A practical guide to single-cell RNA-sequencing for biomedical research and clinical applications. Genome Med..

[9] Fu Y, Huang X, Zhang P, van de Leemput J, Han Z (2020). Single-cell RNA sequencing identifies novel cell types in Drosophila blood. J. Genet. Genomics.

[10] Wolf FA, Angerer P, Theis FJ (2018). SCANPY: Large-scale single-cell gene expression data analysis. Genome Biol..

[11] Phipson B (2022). Propeller: Testing for differences in cell type proportions in single cell data. Bioinformatics.

[12] Cao Y (2019). scDC: Single cell differential composition analysis. BMC Bioinform..

[13] Smillie CS (2019). Intra- and inter-cellular rewiring of the human colon during ulcerative colitis. Cell.

[14] Mangiola S (2023). sccomp: Robust differential composition and variability analysis for single-cell data. Proc. Natl. Acad. Sci..

[15] Büttner M, Ostner J, Müller CL, Theis FJ, Schubert B (2021). scCODA is a Bayesian model for compositional single-cell data analysis. Nat. Commun..

[16] Dann E, Henderson NC, Teichmann SA, Morgan MD, Marioni JC (2022). Differential abundance testing on single-cell data using k-nearest neighbor graphs. Nat. Biotechnol..

[17] Virshup I (2023). The scverse project provides a computational ecosystem for single-cell omics data analysis. Nat. Biotechnol..

[18] Huang Z (2021). Effects of sex and aging on the immune cell landscape as assessed by single-cell transcriptomic analysis. Proc. Natl. Acad. Sci..

[19] Sim CB (2021). Sex-specific control of human heart maturation by the progesterone receptor. Circulation.

[20] Chen ST (2022). A shift in lung macrophage composition is associated with COVID-19 severity and recovery. Sci. Transl. Med..

[21] Regev A (2017). The human cell atlas. ELife.

[22] Kuppe C (2022). Spatial multi-omic map of human myocardial infarction. Nature.

[23] Domcke S (2020). A human cell atlas of fetal chromatin accessibility. Science.

[24] Tirosh I (2016). Dissecting the multicellular ecosystem of metastatic melanoma by single-cell RNA-seq. Science.

[25] Aran D, Hu Z, Butte AJ (2017). xCell: Digitally portraying the tissue cellular heterogeneity landscape. Genome Biol..

[26] Newman AM (2019). Determining cell type abundance and expression from bulk tissues with digital cytometry. Nat. Biotechnol..

[27] Li H (2020). DeconPeaker, a deconvolution model to identify cell types based on chromatin accessibility in ATAC-seq data of mixture samples. Front. Genet..

[28] Kiselev VY, Andrews TS, Hemberg M (2019). Challenges in unsupervised clustering of single-cell RNA-seq data. Nat. Rev. Genet..

[29] Simmons, S. *Cell Type Composition Analysis: Comparison of Statistical Methods*. 10.1101/2022.02.04.479123 (2022).

[30] Ritchie ME (2015). limma powers differential expression analyses for RNA-sequencing and microarray studies. Nucleic Acids Res..

[31] Seabold, S. & Perktold, J. Statsmodels: Econometric and statistical modeling with Python. in *Proceedings of 9th Python Science Conference* (2010).

[32] Smyth GK (2004). Linear models and empirical Bayes methods for assessing differential expression in microarray experiments. Stat. Appl. Genet. Mol. Biol..

[33] Benjamini Y, Hochberg Y (1995). Controlling the false discovery rate: A practical and powerful approach to multiple testing. J. R. Stat. Soc. Ser. B.

[34] Marshall A, Altman DG, Holder RL, Royston P (2009). Combining estimates of interest in prognostic modelling studies after multiple imputation: Current practice and guidelines. BMC Med. Res. Methodol..

[35] Bolt MA (2022). Inference following multiple imputation for generalized additive models: An investigation of the median p-value rule with applications to the Pulmonary Hypertension Association Registry and Colorado COVID-19 hospitalization data. BMC Med. Res. Methodol..

[36] Eekhout I, van de Wiel MA, Heymans MW (2017). Methods for significance testing of categorical covariates in logistic regression models after multiple imputation: Power and applicability analysis. BMC Med. Res. Methodol..

[37] Waskom ML (2021). seaborn: Statistical data visualization. J. Open Source Softw..

[38] Charlier F (2022). Zenodo.

[39] (2024). github.

