# Supplementary material for: Scanpro is a tool for robust proportion analysis of single-cell resolution data
Source: Sci Rep. 2024 Jul 6;14:15581. doi: 10.1038/s41598-024-66381-7 (PMC11227528; doi:10.1038/s41598-024-66381-7)
Supplement: Supplementary file 1 — Supplementary Information. [file 41598_2024_66381_MOESM1_ESM.docx]

Supplementary information

Scanpro is a tool for robust proportion analysis of single cell resolution data

Yousef Alayoubi, Mette Bentsen, Mario Looso

Content

[**Supplementary figures 2**](#_805cm993pdij)

[Supplementary Figure 1: Choice of FDR for scCODA analysis 2](#_h7sxnfwmz8fd)

[Supplementary Figure 2: Proportions of cells per sample across clusters 3](#_luum3y2x796d)

[Supplementary Figure 3: Comparison of Scanpro with propeller and scCODA for heart development data 4](#_g8r9sdm2jy8l)

[Supplementary Figure 4: Scanpro results on COVID-19 data 5](#_pbtk74v505os)

[Supplementary Figure 5: Scanpro results on unreplicated COVID-19 data using pseudo-replicates 6](#_abeq8f6tcril)

[Supplementary Figure 6: Simulation of data for benchmarking 7](#_t6soudjblhqr)

[Supplementary Figure 7: Percent correct assignments for Scanpro runs 8](#_pye6q2eybzm1)

[Supplementary Figure 8: Benchmarking unreplicated datasets with scCODA 9](#_ua2s7zlaa60e)

[Supplementary Figure 9: Human myocardial infarction atlas cell type proportions 10](#_l7br512wuxtf)

[Supplementary Figure 10: Human myocardial infarction atlas cell type proportions using pseudo-replicates 11](#_dullpx48lknt)

[Supplementary Figure 11: Fetal scATAC atlas cell type proportions for all tissues 12](#_k5xnmxuynles)

[Supplementary Figure 12: Scanpro analysis on PBMC data with covariates 13](#_yiasi0b1ka9u)

[**Supplementary methods 14**](#_m1o5s3vq9gee)

[Scanpro software architecture 14](#_gym94svqaoiu)

[Transform Proportions 14](#_gu3cy9439auj)

[Empirical Bayes statistics 14](#_krq79qojaqo3)

[Bootstrapping method to simulate replicates for unreplicated data 15](#_3klyd4rxmajw)

# Supplementary figures


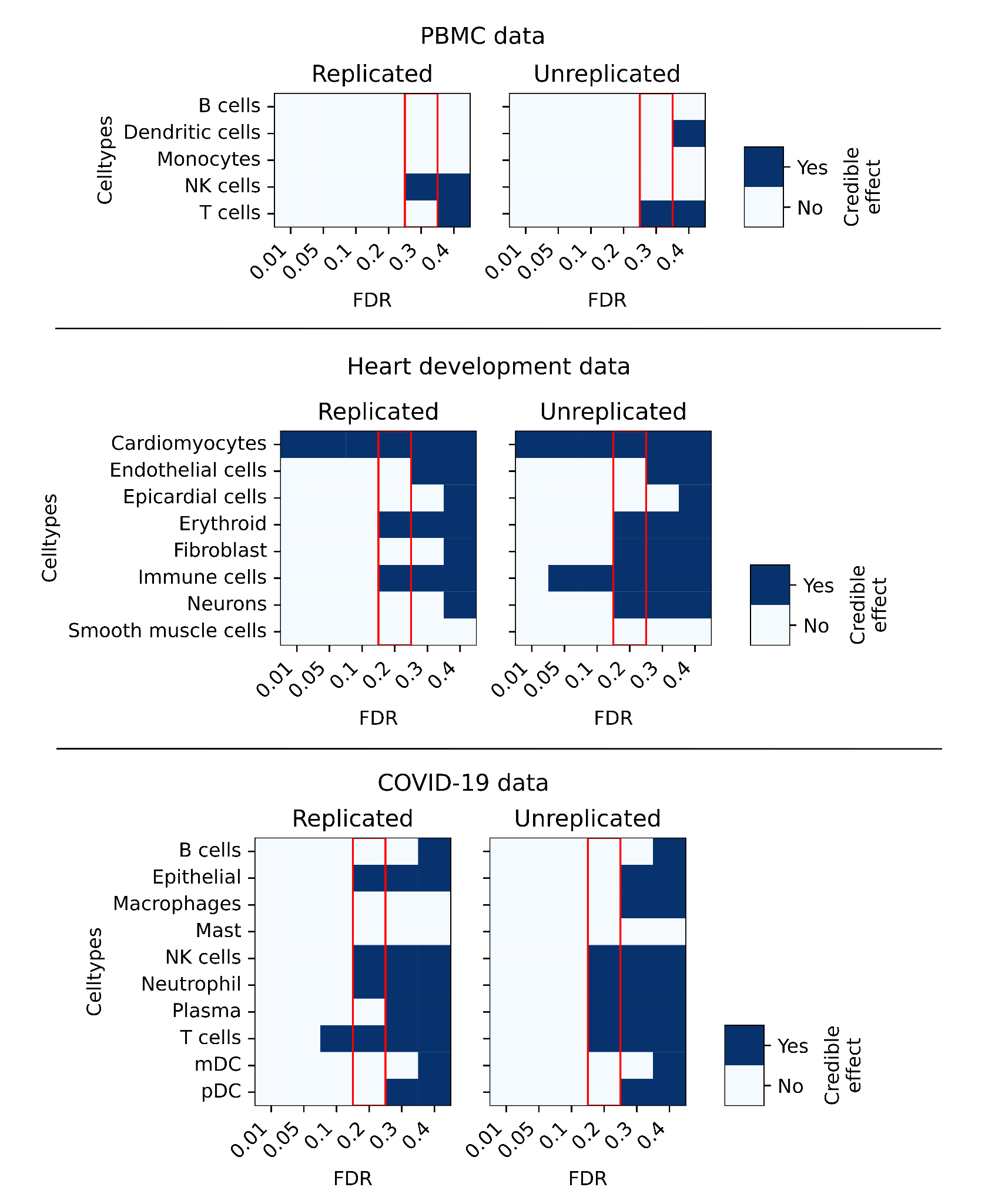


## **Supplementary Figure 1: Choice of FDR for scCODA analysis**

scCODA was run for all three datasets with increasing FDR and the credible effect of each cluster was estimated at each FDR level. Red blocks indicate the chosen FDR level.


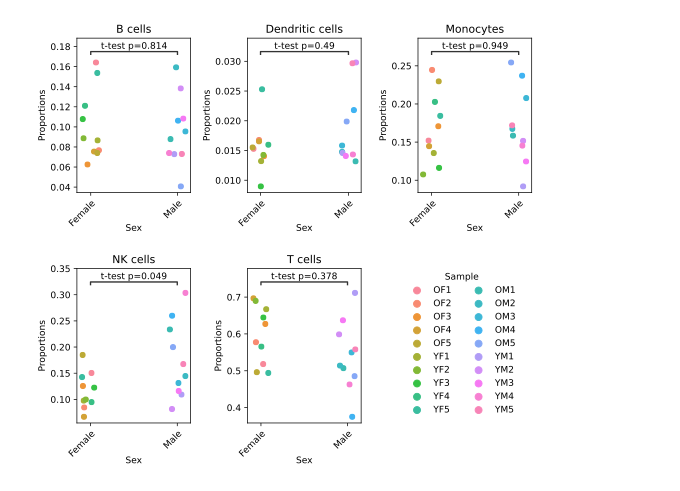


## **Supplementary Figure 2: Proportions of cells per sample across clusters**

Output from Scanpro showing the raw proportions of cells from the PBMC dataset in each cluster. The samples are split between male and female samples per cluster. OF = Old female; YF = Young female; OM = Old male; YM = Young male. The p-values are calculated by Scanpro using “logit” transformed data.


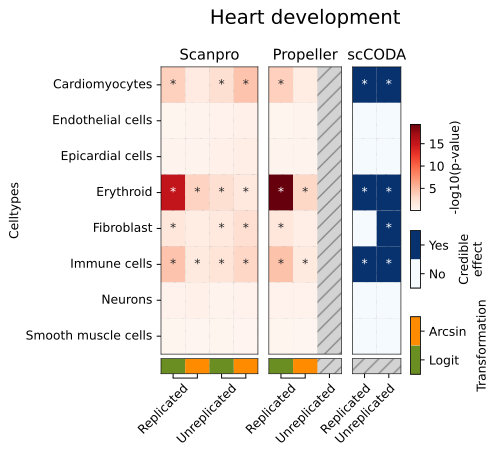


## **Supplementary Figure 3: Comparison of Scanpro with propeller and scCODA for heart development data**

-log10(p-values) were calculated for scanpro and propeller (red color in the heatmap). For scCODA, credible effects are plotted. Asterisks denote significant changes (p-value < 0.05 or credible effect=TRUE). For scCODA, default settings were used.

##
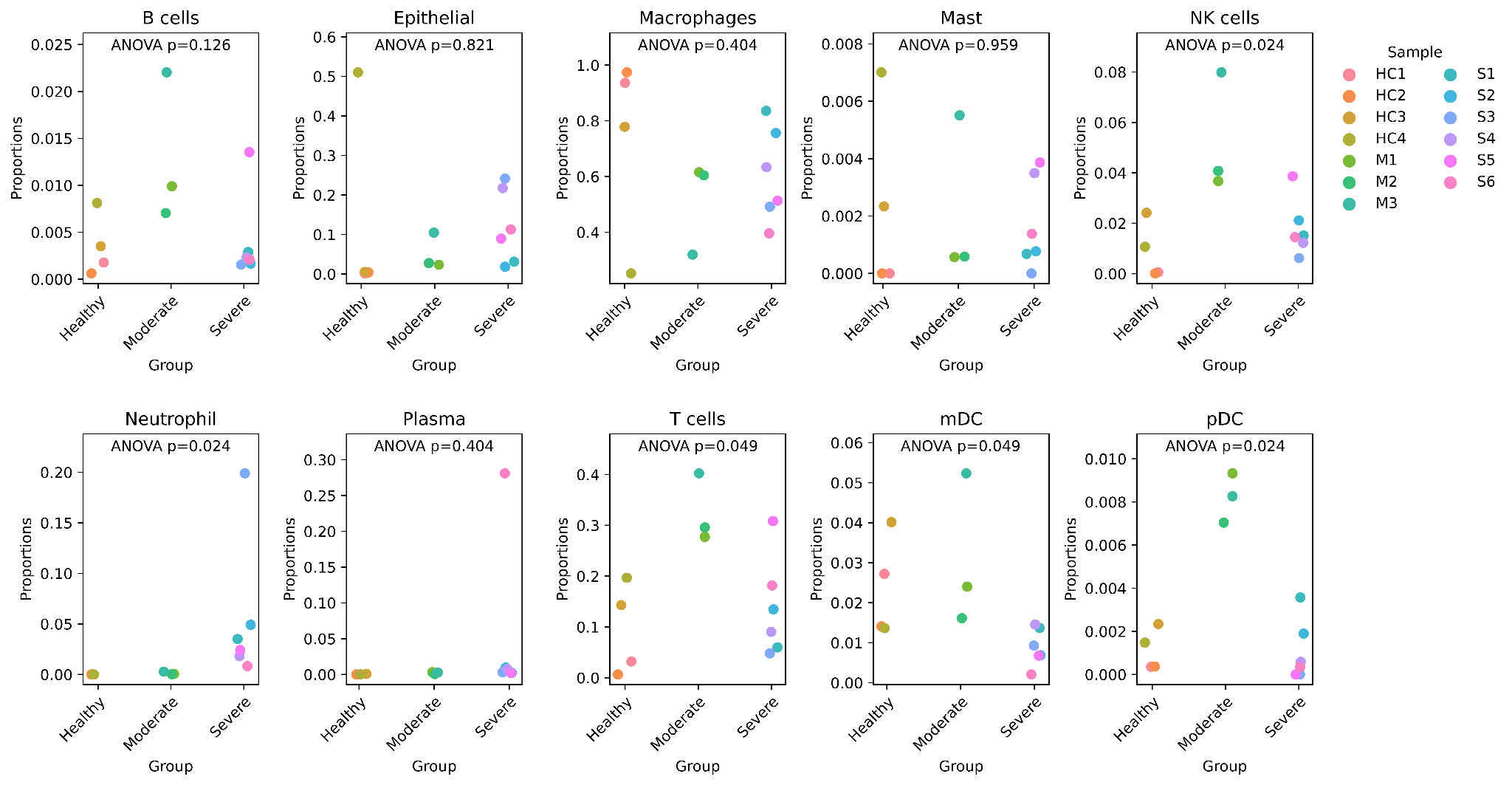


## **Supplementary Figure 4: Scanpro results on COVID-19 data**

Output from Scanpro showing the proportion of each cell type per sample. p-values represent ANOVA comparison of Healthy, Moderate and Severe groups with arcsin transformation. HC = Healthy control; M = Moderate; S = Severe.

##
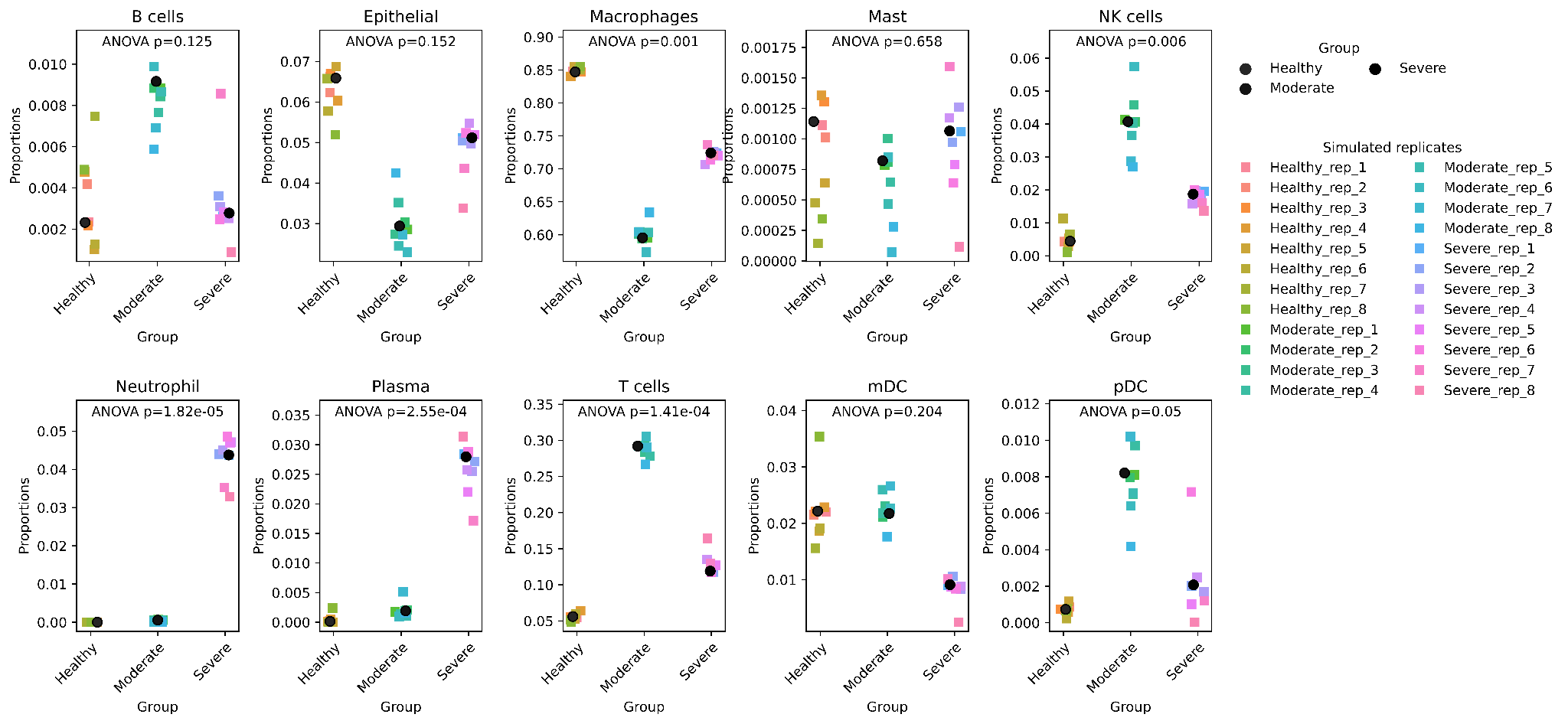


## **Supplementary Figure 5: Scanpro results on unreplicated COVID-19 data using pseudo-replicates**

Stripplots show the proportion of each cell type per sample. The group means are plotted in black circles. The colored squares represent simulated replicates per group/cluster. p-value is calculated in arcsin transformed data.

##

##


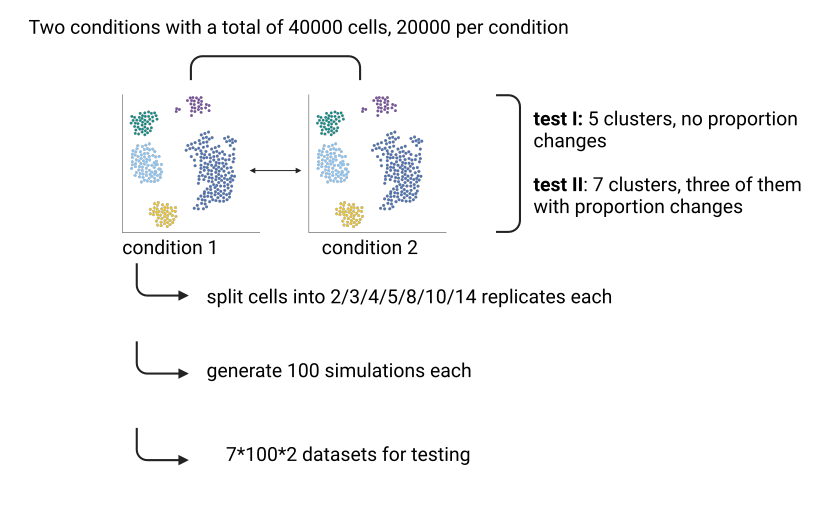


## **Supplementary Figure 6: Simulation of data for benchmarking**

For each number of replicates, 100 datasets were generated. Each condition contains a total of 20000 cells, meaning that the number of cells per sample is the total divided by the number of replicates. 100 datasets were simulated for each of the 7 numbers of replicates, equaling 700 datasets per test case (total of 1400 datasets).


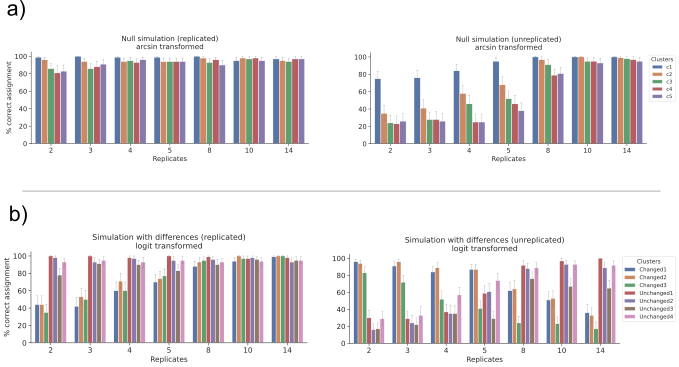


## **Supplementary Figure 7: Percent correct assignments for Scanpro runs**

a) Results of a null simulation where none of the clusters are significantly changed. Left shows replicated data, right shows unreplicated data. b) Results of a simulation with differences in three of seven clusters with logit transformation. Left shows replicated data, right shows unreplicated data. Lines represent the mean values given by 100 iterations.


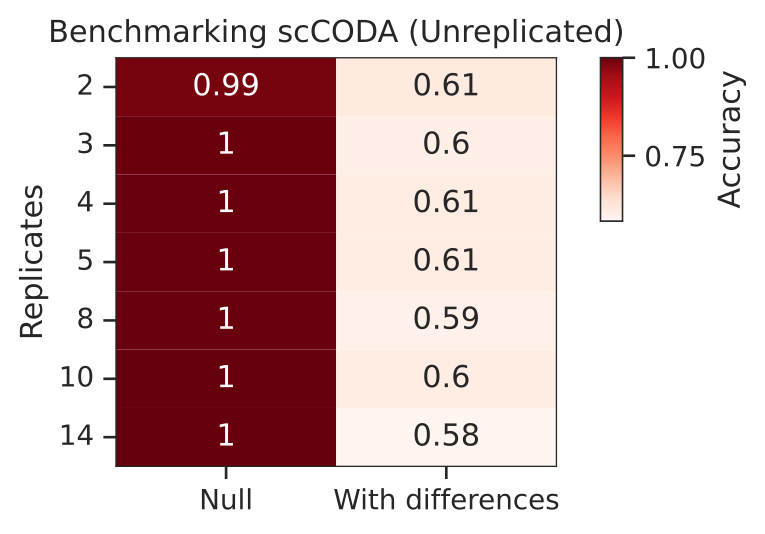


## **Supplementary Figure 8: Benchmarking unreplicated datasets with scCODA**

Left column represents accuracy values for scCODA in unreplicated mode on the null simulation. Right column indicates accuracy values for the three out of seven with differences simulation. Number of replicates refers to simulated replicates per condition before merging them into one per condition. FDR for scCODA was set to 0.05 to be comparable with the significance level for Scanpro (p=0.05).


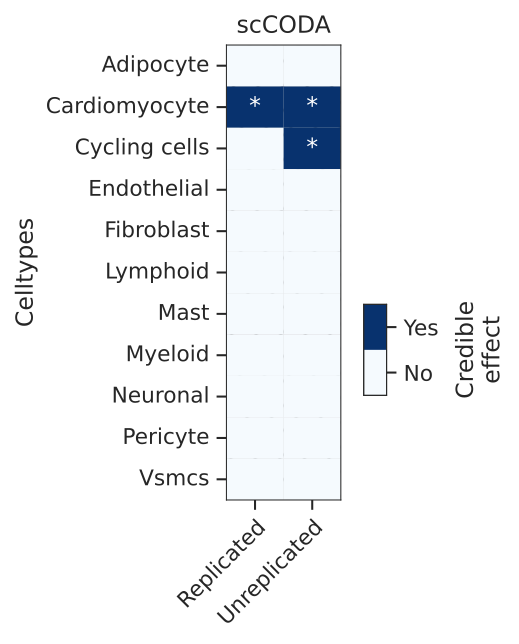


## **Supplementary Figure 9: scCODA results for Human myocardial infarction atlas data**

Utilizing all control and ischaemic cells as replicates (left) and joined (right), Credible changes in cell proportions detected by scCODA with FDR=0.05.

##
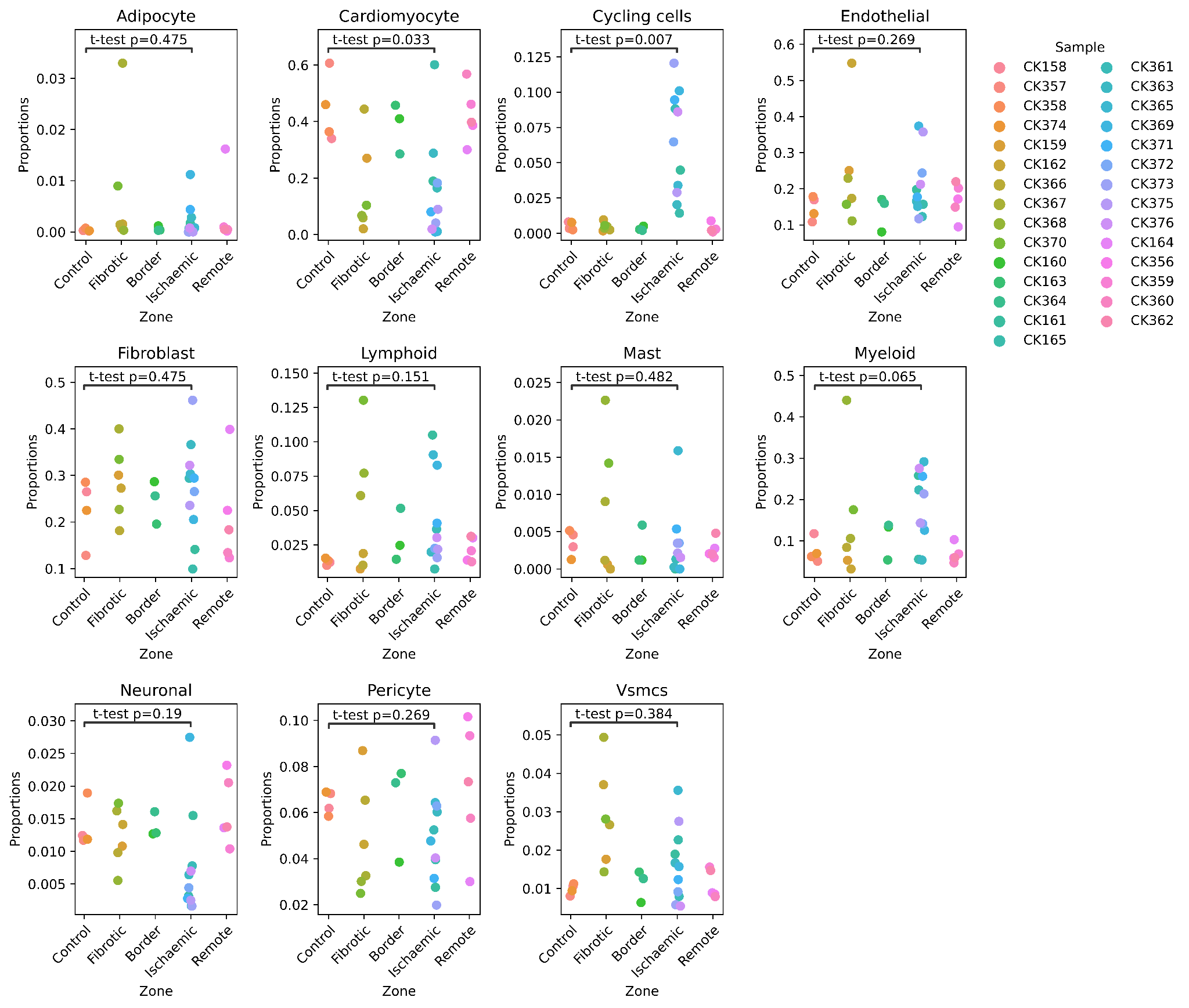


## **Supplementary Figure 10: Human myocardial infarction atlas cell type proportions**

Scanpro stripplots showing the proportion of cell types within the original samples. p-values are calculated between control and Ischaemic groups with arcsin transformation.

##
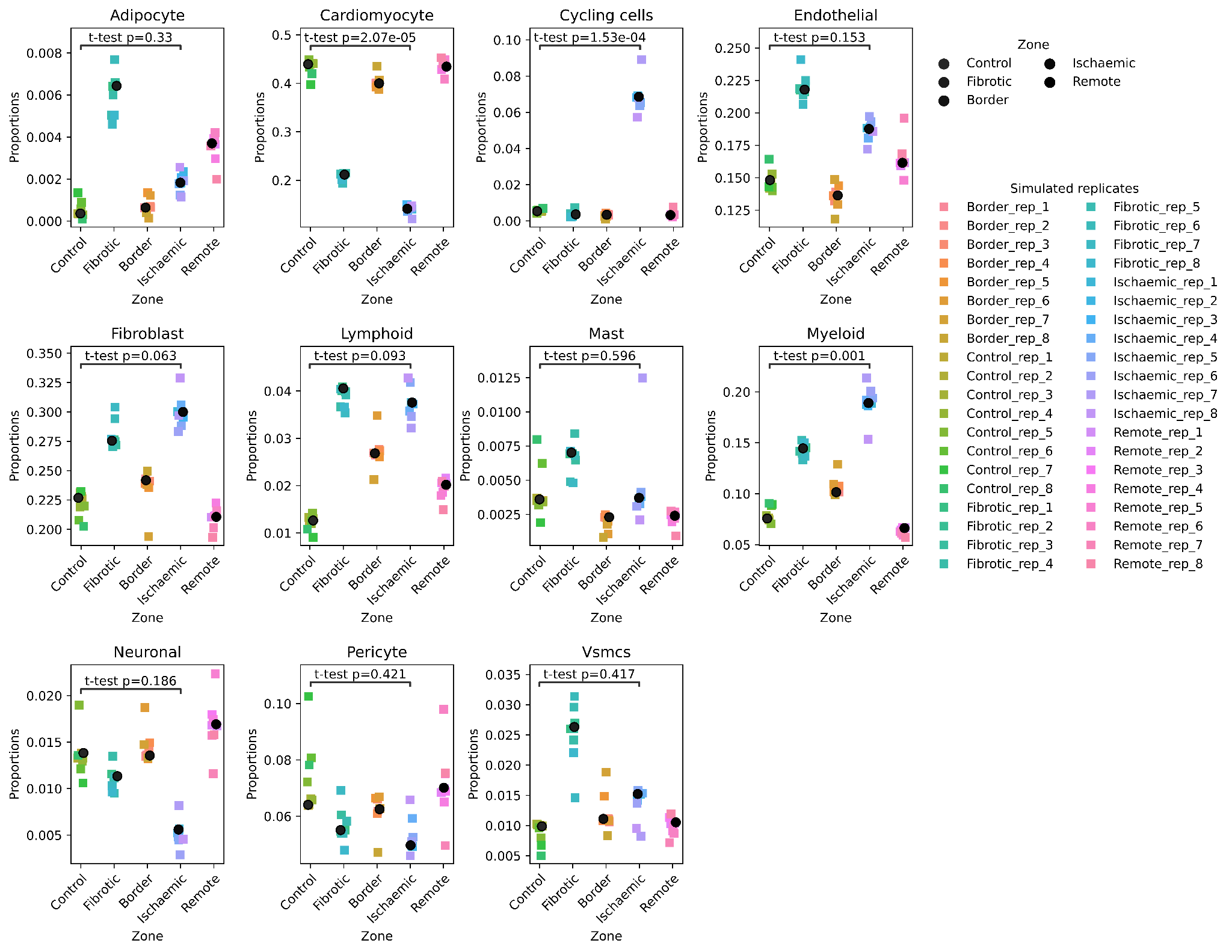


## **Supplementary Figure 11: Human myocardial infarction atlas cell type proportions using pseudo-replicates**

Stripplots show the proportion of each cell type per sample. The group means are plotted in black circles. The colored squares represent simulated replicates per group/cluster. p-value is calculated in arcsin transformed data.

##

#

#

#


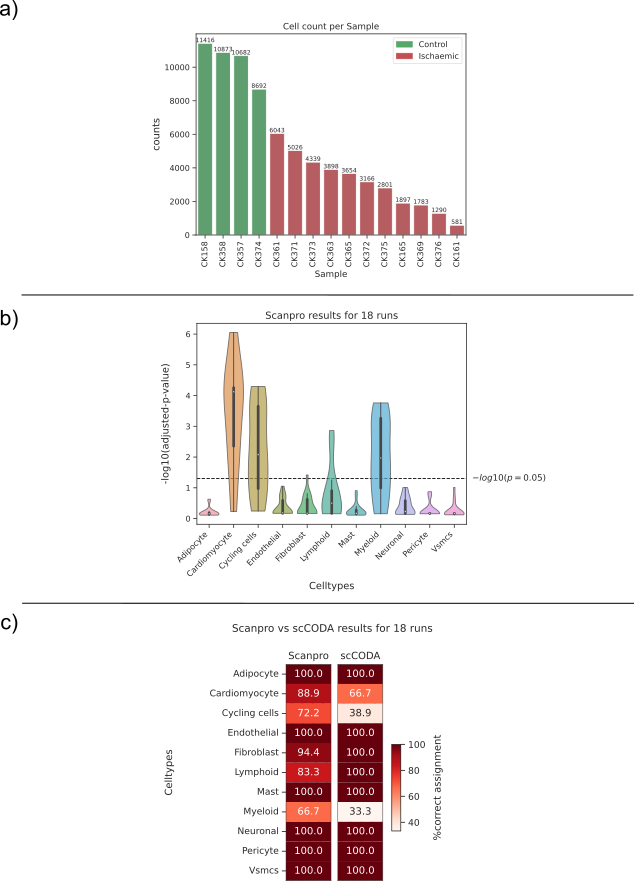


## **Supplementary Figure 12: Scanpro and scCODA results for individual replicates in Human myocardial infarction atlas**

1. Cell counts and condition per sample from Kuppe et al.
2. We excluded CK161,165,369,375,373, and 373 due to some cell types having 0 count.

CK357 was excluded due to the relatively high count of cardiomyocytes (*n=6478*). Each pairwise combination of one control and ischemic sample (*total = 18*) was tested individually with scanpro ; p-values on y-axis were adjusted using benjamini-hochberg method and log-normalized

1. Result comparison between scanpro and scCODA for all 18 runs. Heatmap shows correct assignment of each cluster in 18 runs, assuming cardiomyocyte, cycling cells and myeloid proportions to change significantly.

## **
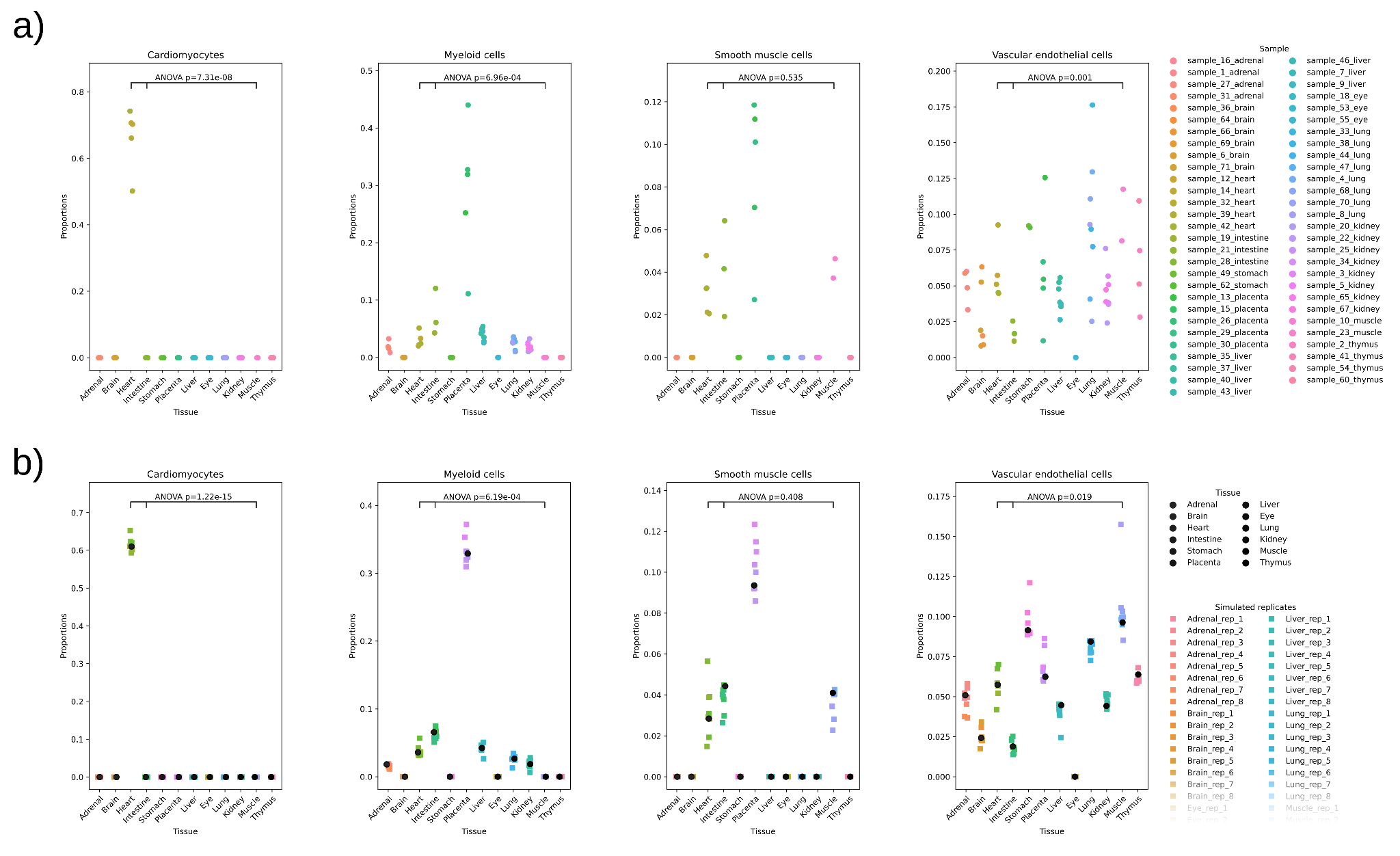
**

## **Supplementary Figure 13: Fetal scATAC atlas cell type proportions for all tissues**

Stripplots show the proportion of each cell type per sample across all tissues. The p-values are calculated on arcsin transformed data. a) The proportions of original samples. b) The proportions of pseudoreplicates simulated by Scanpro.

##


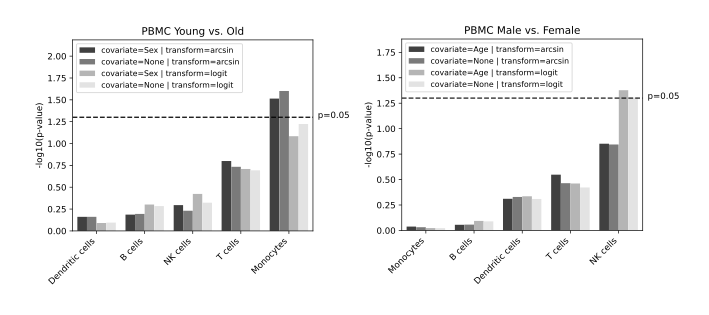


## **Supplementary Figure 14: Scanpro analysis on PBMC data with covariates**

Direct comparison of runs with/without covariates (sex/age) and for transformation (logit/arcsin), indicating -log10 transformed p-values on the Y axis and cell types on the X axis. Left: young vs. old PBMCs; Right: male vs. female PBMCs.

# Supplementary methods

## Scanpro software architecture

### Transform Proportions

The workflow of the {get_transformed_props} function is shown below:

*
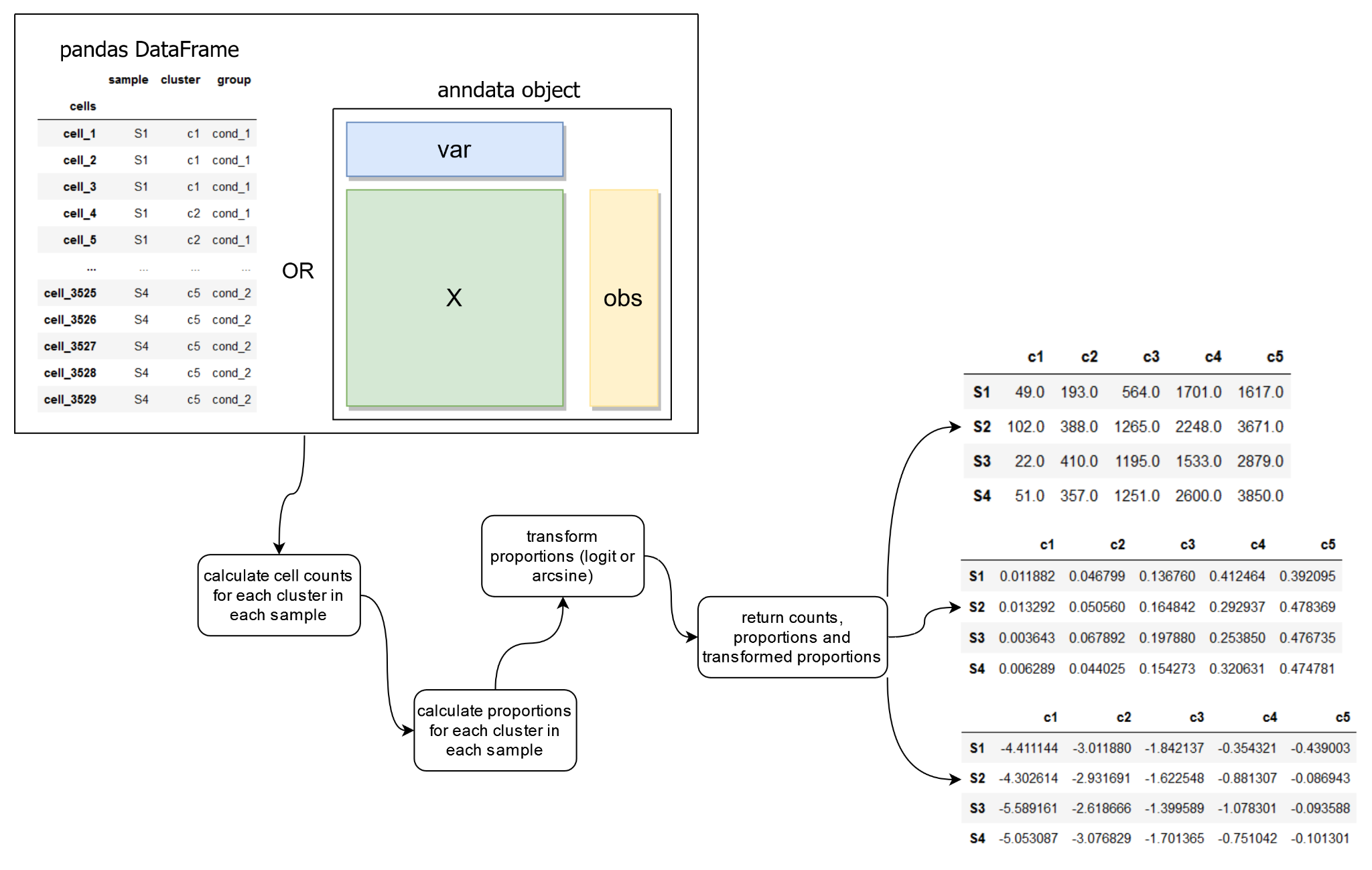
*

The function returns three matrices: counts (top), proportions (middle), and transformed proportions (bottom).

### Empirical Bayes statistics

The empirical bayes method is implemented in scanpro as {ebayes}. To test significance, empirical bayes moderated t-test (for two conditions) and ANOVA (for more than two conditions) are used. Estimated p-values are then adjusted for multiple testing using the Benjamini-Hochberg method. The two functions {t_test} and {anova} are implemented as wrapper functions to perform the linear model fitting and empirical bayes statistics. The function {run_scanpro} is implemented to perform all steps.The function takes a cell proportion matrix and a design matrix as input. The output is p-values and adjusted p-values.

The workflow of the {run_scanpro} function is:


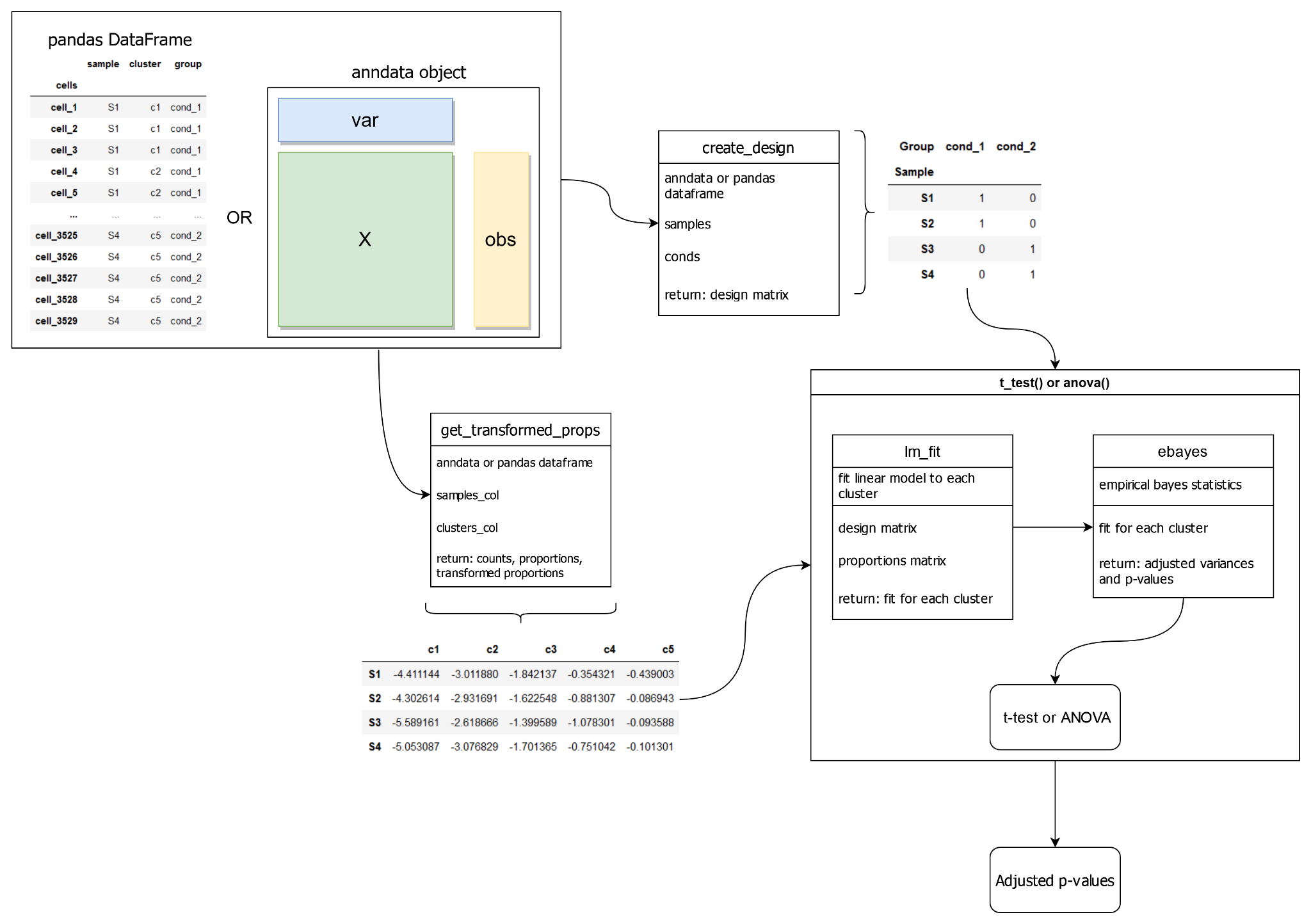


### Bootstrapping method to simulate replicates for unreplicated data

The bootstrapping method to generate replicates is implemented as {generate_reps} function. The function {sim_scanpro} is a wrapper function that performs the simulations. It also allows setting the number of simulations {n_sims} and the number of replicates {n_reps} manually. The default value for {n_sims} is 100 and for {n_reps} is 8. The bootstrapping without replacement method generates pseudo-replicates for each sample to run the scanpro method. The workflow of the {generate_reps} function is as follows:

*
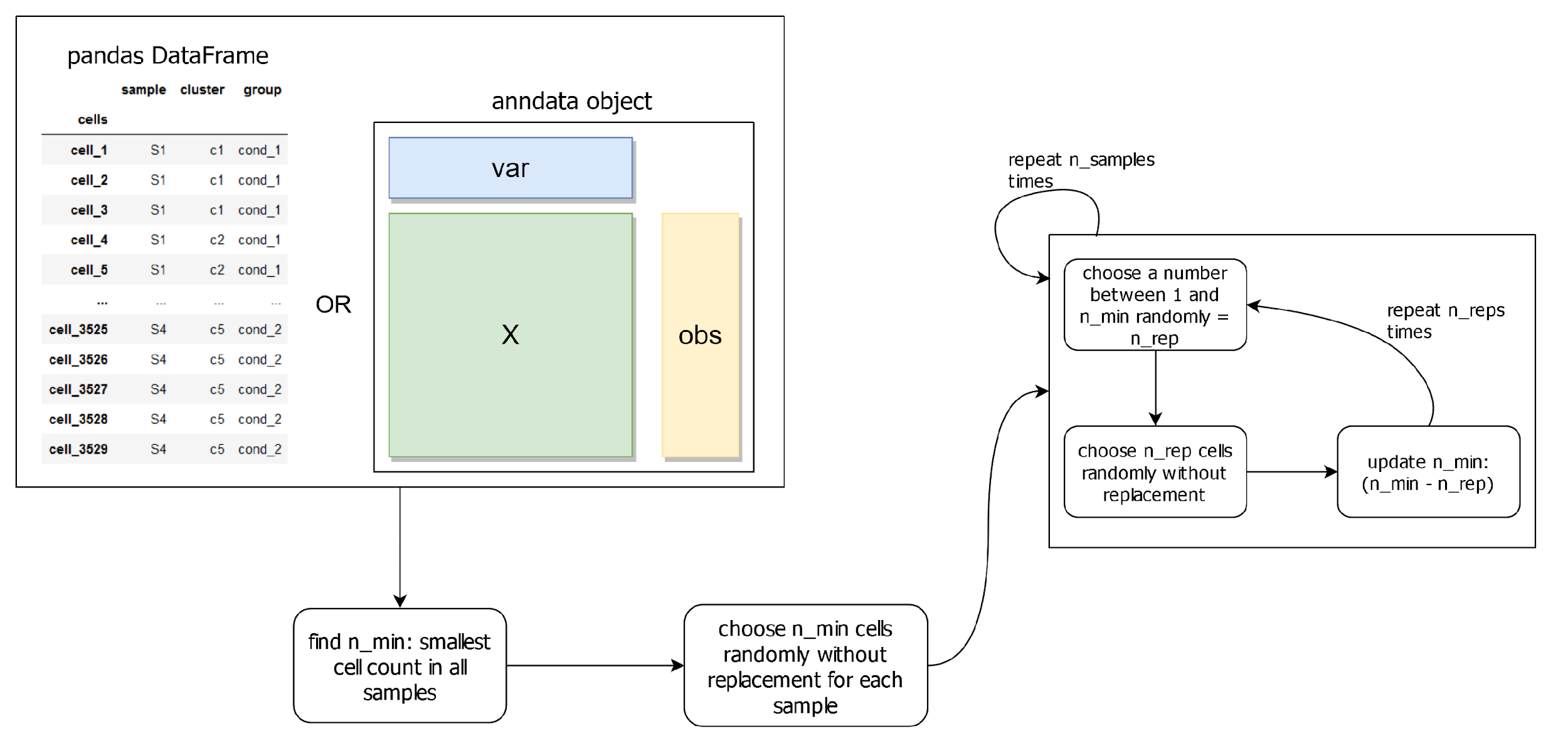
*

The adjusted p-values from {n_sims} runs are collected and the median is calculated as a final result:

*
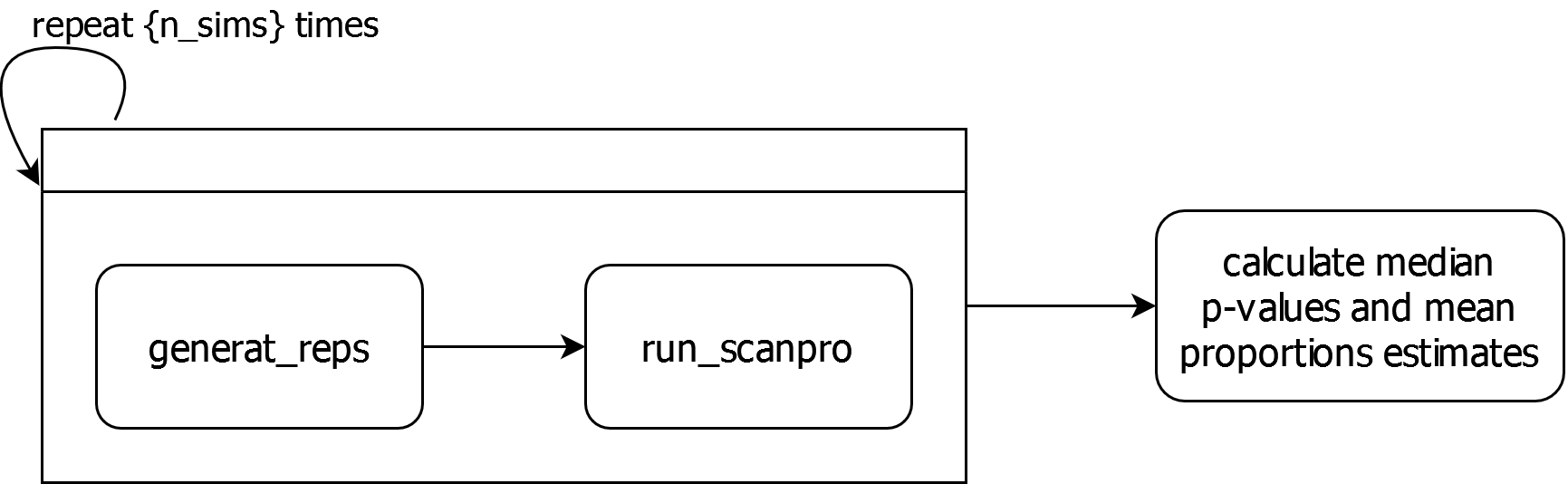
*
